# Biological and catalytic potential of sustainable low and high valent metal-Schiff base sulfonate salicylidene pincer complexes†

**DOI:** 10.1039/c9ra06816c

**Published:** 2019-10-25

**Authors:** Mohamed Shaker S. Adam, Omar M. El-Hady, Farman Ullah

**Affiliations:** Department of Chemistry, College of Science, King Faisal University P. O. Box 380, Al Hofuf Al Ahsa 31982 Saudi Arabia madam@kfu.edu.sa shakeradam61@yahoo.com; Chemistry Department, Faculty of Science, Sohag University Sohag-82534 Egypt; Department of Chemistry, Winnipeg University 515 Portage Avenue Winnipeg Manitoba R3B 2E9 Canada

## Abstract

ONO-Pincer Schiff base salicylidene (HSaln ligand) complexes with VO^2+^, UO_2_^2+^, MoO_2_^2+^ and Mn^2+^ ions (MSaln complexes = VOSaln, UO_2_Saln, MoO_2_Saln and MnSaln, respectively) were synthesized and fully characterized by different physico-chemical tools. The VOSaln complex was further treated with 1,10-phenanthroline which afforded a new VO-complex (VOSaln-Ph). All complexes and their ligands, as eco-friendly reagents, were explored for their biological potential as antibacterial and antifungal agents. Reactivity of MSaln complexes against the tested pathogen strains exhibited a remarkable inhibitory effect compared to the coordinated ligand (HSaln) and applicable standard drugs. Moreover, the MSaln complex-DNA interaction was investigated by ultraviolet-visible spectroscopy, viscosity and gel electrophoresis techniques affording binding strengths in the order: UO_2_Saln > MnSaln > MoO_2_Saln > VOSaln-Ph > VOSaln. Additionally, the biological potential of the investigated compounds was further explored by molecular docking to illustrate the nature of the drug–DNA interactions. All MSaln complexes show respectable anti-proliferative potential as anticancer agents against selected human carcinoma cell lines. Aside from the biological activities these complexes (MSaln complexes) were also investigated for catalytic efficiency in the Suzuki–Miyaura cross-coupling system of phenylboronic acid with 2-bromopyridine in water, sustainably. The results indicated that the MnSaln catalyst performed well with high yield. The catalytic potential of MnSaln was compared in water, water–ionic liquid mixtures and ionic liquids.

## Abstract 2

ONO-Tridentate Schiff base complexes with VO^2+^, UO_2_^2+^, MoO_2_^2+^ and Mn^2+^ were synthesized and characterized. All the complexes were shown to be of potential use as anticancer agents against selected human carcinoma cell lines.

## Graphical abstract

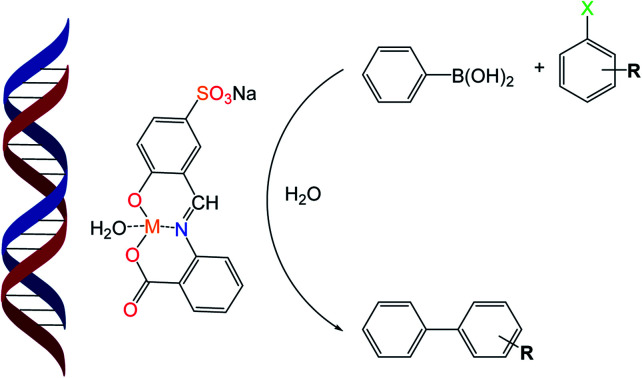

## 1. Introduction

Schiff bases, as azomethine or imine derivatives (–CH=N–), are the most famous chelating ligands for complexation with transition metal ions of different oxidation states to afford highly stable metal pincer complexes.^1,2^ Wide applicability of M-Schiff base pincer chelates could be observed in many areas, *e.g.* biological (antibacterial, antifungal, antiviral and anti-inflammatory effectiveness),^3–5^ pharmaceutical,^6^ catalysts,^7–11^ sensors,^12^ and organic photovoltaic materials.^13^ The indispensable function of the metal ion and its charge in the Schiff base complexes could help to measure their potential biologically, *e.g.* with DNA cleavage.^14,15^ The structural features of oxy- or dioxy-vanadium Schiff base chelates^16,17^ are of interest biologically,^18^ as an inhibitor of protein-tyrosine phosphatase 1B^19^ and DNA interacting reagents, as reported in the literature.^15,20^ Some biological studies of vanadyl Schiff bases and other metal Schiff base complexes have been reported recently by Rudbari *et al.*^21^ and by Shah *et al.*,^22^ respectively.

Cancer remains the main cause of death globally. The International Agency for Research on Cancer (IARC) recently reported that 7.6 million deaths globally were due to cancer with millions of new cases per year being reported worldwide. With continues growth of cancer patients globally pushing the scientific community to discover new drugs with minimum side effects. Many research groups are focused to overcome the massive chemotherapeutic drugs side effects, as metal-complex drugs.^14,20^ Hence, many reported works highlighted on the research for the development of various metal Schiff base chelates as anticancer agents along with the study of their side effects.^23^ DNA binding and the anticancer reactivity on transition metal Schiff base complexes have been evaluated recently and reported in details, *e.g.* by Abdel-Rahman *et al.*^24,25^

The high reactivity of M-Schiff base pincer chelates towards DNA play pivotal role for such progressing in nucleic acids biochemically as foot-printing reagents, models for limited enzymes and therapeutic use as drugs.^26^ Currently, there are many published literatures reporting the anticancer activities of various transition metal Schiff base complexes, Abd El-Halim *et al.*^27^ studied the Cr^2+^, Fe^2+^, Mn^2+^, Cu^2+^, Cd^2+^, Co^2+^ and Ni^2+^-1,10-phenanthroline–Schiff base complex as high antimicrobial and anticancer reagents. The antimicrobial and anticancer activity of Bi^3+^, Pb^2+^, Cr^3+^, Cu^2+^, Cd^2+^, Ba^2+^, Ni^2+^, Fe^2+^, Nd^2+^ and Sn^2+^–Schiff base complexes are studied by Sheng *et al.*^28^ and Sharaby *et al.*^29^ They investigated the microbial and anticancer potentials of sulfonamide imine and mixed coordinated ligand complexes with glycine spectroscopically. Mahmoud *et al.* published many biological studies of bidentate Schiff base ferrocene metal complexes derived from amino acids.^30–33^ Manganese and some other metal chelates of polymeric Schiff base ligand were studied as antimicrobial reagents by Rasool and Hasnain.^34^ Moreover, the coordination chemical behaviour and biological activity of Zn^2+^, Cu^2+^, Ni^2+^, Co^2+^ and Mn^2+^ towards ONO-tridentate imine ligand, *i.e.* 2-[(2-hydroxy-naphthalen-1-ylmethylene)-amino]-benzoic acid, as a non-water-soluble ligand was investigated and reported by Refat *et al.*^35^ Zayed *et al.* presented the coordination chemistry, biological efficiency and molecular docking studies of various transition metal complex.^36–39^ All previous biological reported studies assigned important results for the high potential of M-Schiff base pincer chelates towards bacteria, fungi and cancers, as well as, high DNA-cleavages.

C–C, C–O and C–N cross-coupling protocols are one of the most famous catalytic organic approaches for various organic syntheses.^40^ High oxidation state metal complexes are widely known as catalysts for oxidation and oxygenation processes, *e.g.* MoO_2_^2+^, WO_2_^2+^, UO_2_^2+^, VO^2+^ and VO^+^ species.^20,41–43^ Low valent metal complexes, *e.g.* M^2+^ ions, are highly active catalysts for most of the cross couplings^44,45^ in order to generate M^0^ active catalysts. For example, Mn^2+^-pincer chelates display an effective role as homogeneous catalysts for most versatile and prevalent recent synthetic devises under sustainable conditions.^46^ Sustainably, the incorporated sodium sulfonate group to the synthesized Schiff base ligand improves its reactivity and the corresponding metal complexes under green conditions.^41^ Ionic liquids, as imidazolium- and pyridinium-based liquids are of interest as solvents^47–50^ or co-catalysts^51–54^ for many C–C biaryls syntheses, *e.g.* Suzuki–Miyaura cross-coupling protocols.

In the present work, we present the synthesis, biological activities and catalytic performance of the polar chelate pincer complexes, synthesized from the water-soluble Schiff base pincer ligand with various metal ions, *i.e.* MoO_2_^2+^, UO_2_^2+^ and VO^2+^ and Mn^2+^ ions. Their biological reactivity was investigated as antimicrobial and anticancer agents. Moreover, DNA interaction with the prepared complexes was explored and supported by the molecular docking studies. We also report the catalytic behavior with the high valent metal ions in their Schiff base complex catalysts, *i.e.* MoO_2_^2+^, UO_2_^2+^ and VO^2+^ ions, as well as, Mn^2+^-complex in Suzuki–Miyaura cross-coupling processes of arylboronic acid with different aryl halides, representatively.

## 2. Results and discussion

### 2.1. Synthesis and structure estimation

**Scheme 1:**
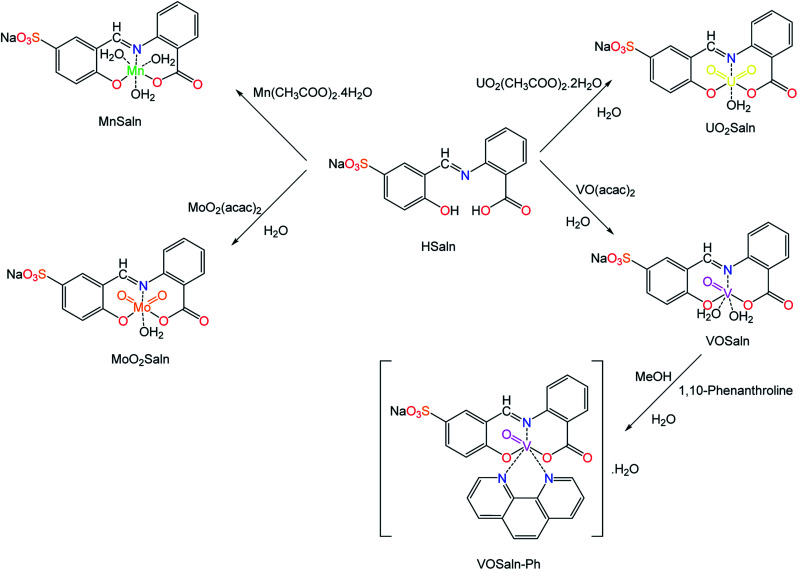
Diagrammatic scheme for the synthesis of MSaln complexes from HSaln ligand.

ONO-Tridentate polar pincer ligand (HSaln ligand) was prepared according to the well-known condensation reaction protocol, which was obtained from the reaction of 1.0 equivalent of anthranilic acid in ethanol with 1.0 equivalent of 5-salicylaldehyde-sodium sulfonate sustainably.^41^ HSaln ligand was fully characterized by the most valuable spectroscopic techniques (Tables 1 and 2, which are supported by attached files in the ESI†). Moreover, the coordination features of HSaln ligand were investigated by complexation with M^2+^ ions in an aqueous media.^41^ Hence, a new series of complexes with metal ions of high and low oxidation states, *i.e.* with VO^2+^, MoO_2_^2+^, UO_2_^2+^ and Mn^2+^ ions as MSaln complexes were synthesized. All MSaln complexes were prepared under sustainable conditions, *i.e.* in H_2_O, with 1 : 1 ligand metal ions ratio afforded VOSaln, MoO_2_Saln, UO_2_Saln and MnSaln, respectively (Scheme 1). VOSaln in ethanol was further treated with 1,10-phenanthroline (1 : 1 ratio) provided a new complex (VOSaln-Ph) by substitution of the two coordinated H_2_O molecules as observed elsewhere.^43^ To avoid oxidation of Mn^2+^ ions to Mn^3+^ ion in the synthetic pathway of MnSlan, the reaction carried out under N_2_ atmosphere.^46^

**Table 1:** Distinctive elemental analysis, melting point, color, electronic and mass spectra (electrospray ionization mass spectra) HSaln ligand and its MSaln complexes at [compound] = 1 × 10^−5^ mol dm^−3^ in H_2_O at 25 °C

Compound	MW (g mol^−1^)	Elemental analysis found%, (calc.%)			Color	Mp (°C)	Electronic transition spectra			Mass spectra (*m*/*z*)	
		C	H	N			*λ*_max_ (nm)	*ε*_max_ (mol^−1^ cm^−1^)	Assign.	[M + Na^+^]	[M − Na^+^]
HSaln ligand	343.29	49.25 (48.98)	3.08 (2.94)	3.81 (4.08)	Pale yellow	164	325, 256	5894, 12 436	n → π*, π → π*		
VOSaln	444.24	38.11 (37.85)	2.98 (2.72)	3.07 (3.15)	Dark green	288	395, 310, 249	5073, 8211, 12 099	LM-CT, n → π*, π → π*	465, 467, 468, 469, 471	419, 420, 421, 422, 423
VOSaln-Ph.·H_2_O	606.43	51.76 (51.50)	3.02 (2.99)	7.36 (6.93)	Brownish green	>300	376, 322	4863, 8956	LM-CT, n → π*	610, 611, 612	565, 566
UO_2_Saln	629.31	27.10 (26.72)	1.94 (1.60)	2.12 (2.23)	Deep yellow	304	388, 299, 271, 238	3418, 5874, 6693, 10 112	LM-CT, n → π*, π → π*, π → π*	604, 605, 606, 607	651, 652, 653
MnSaln	250.25	37.47 (37.35)	3.44 (3.13)	2.86 (3.11)	Brown	294	416, 329, 284	3015, 7488, 18 894	LM-CT, n → π*, π → π*	471, 473, 474	425, 427, 428
MoO_2_Saln	287.24	34.66 (34.51)	1.77 (2.07)	2.53 (2.87)	Orange	>300	449, 320, 262	2322, 6152, 15 749	LM-CT, n → π*, π → π*	4645, 465	510, 511, 512

**Table 2:** Specific infrared spectral assignments (ν̄, cm^−1^), distinguished magnetic moments (*μ*) and molar conductivity values (*Λ*_m_) of HSaln ligand and its MSaln complexes ([compound] = 1 × 10^−3^ mol dm^−3^) in DMSO and DMF at ambient temperature^a^

Group		Compound					
		HSaln ligand	VOSaln	VOSaln-Ph	UO_2_Saln	MoO_2_Saln	MnSaln
O–H_(water)_			3420(m br), 3342(m br)	3368(m br)	3403(w), 3135(w br)	3352(w br), 3296(m br)	3308(m br), 3233(m br)
O–H		3401(w br)					
C–H_ar_		3058(w)	3062(w)	3080(w)	3074(w br)	3124(w)	
C=O		1601(m)	1597(s)	1585(s)	1589(m)	1693(m)	1643(m)
C–O_(phenolic)_		1494(w)	1451(m)	1455(m)	1470 (m)	1478(w)	1455(m)
C–O_(carboxylic)_		1419(w)	1422(m)	1410(w)	1409 (w)	1403(m)	
C=N_(azomethine)_		1577(w)	1518(s)	1531(s)	1559(m)	1612(m)	1589(m)
C–N		1236(m)	1175(s)	1154(m)	1159(m)	1162(s)	1164(s)
S–O^−^		1382(w)	1340(m)	1372(m)	1362(m)	1378(w)	1402(m)
S=O		1103(m)	1142(m)	1108(m)	1099(m)	1112(s)	1107(s)
M=O			962(m)	964(m)	900(m)	919(w)	
M–O			722(w)	743(w)	723(m)	749(w)	751(w)
M–N			608(m)	608(m)	666(w)	583(m)	601(w)
*Λ*_m_ (Ω^−1^ cm^2^ mol^−1^)	DMSO	139	140	130	117	125	142
	DMF	158	154	149	142	151	163
*μ* (B.M.)		—	2.79	2.24	—	—	5.74

a br (broad), s (strong), m (moderate), w (weak), ar (aromatic CH), alph (aliphatic CH).

All M-complexes (MSaln) were characterized ^1^H-NMR, ^13^C-NMR, infrared, electronic and mass spectra, elemental analyses, thermogravimetric analyses (TGA), measurements of conductance, and magnetic susceptibilities were utilized to estimate the complexation mode of those metal ions with HSaln ligand.^44^ They are fairly soluble in water due to the presence of a polar group (Na^+^SO_3_^−^–) and in DMSO as high coordinated solvent. They are sparingly soluble in acetone, methanol and acetonitrile. C, H and N percentages values of the elemental analysis, as recorded in Table 1, are very close to the proposed structure of all MSaln complexes, with very little difference up to ±0.4% referring to high purity of all MSaln complexes.

^1^H and ^13^C NMR spectra of HSaln ligand are reported, presented in the ESI†^41^ and compared to those complexes, *i.e.* MoO_2_Saln, and UO_2_Saln. NMR spectra of MoO_2_Saln and UO_2_Saln were measured in DMSO-*d*_6_ at room temperature and presented in Fig. S5–S9 in the ESI.†

In ^1^H NMR spectra, the disappearance of a singlet at 10.26 ppm which belongs to –OH group of the HSaln ligand in MoO_2_Saln and UO_2_Saln is the indication for the de-protonation of hydroxyl and formation of desired complexes.^41^ Similarly, the distinguished –OH of the carboxylic group in HSaln ligand within deprotonation coordinated to M^2+^ ion. The characteristic broad singlet signal of the Schiff base (–CH=N–) was shifted from 10.82 ppm (HSaln ligand) to 11.00 and 9.34 ppm in MoO_2_Saln and UO_2_Saln respectively, by the coordination of nitrogen lone pair in the Schiff base moiety, as observed elsewhere^55^ (Scheme 1). From ^13^C NMR spectral scans, the signal of the Schiff base group (–CH=N–) was clearly observed when shifted after coordination to MoO_2_^2+^ and UO_2_^2+^ ions from 192.20 ppm to 163.41 and 161.20 ppm in MoO_2_Saln and UO_2_Saln, respectively. Moreover, the carboxylate C=O group was little shifted from 169.22 ppm^41^ to 169.92 and 174.11 ppm in MoO_2_Saln and UO_2_Saln, respectively.

**Fig. 1:**
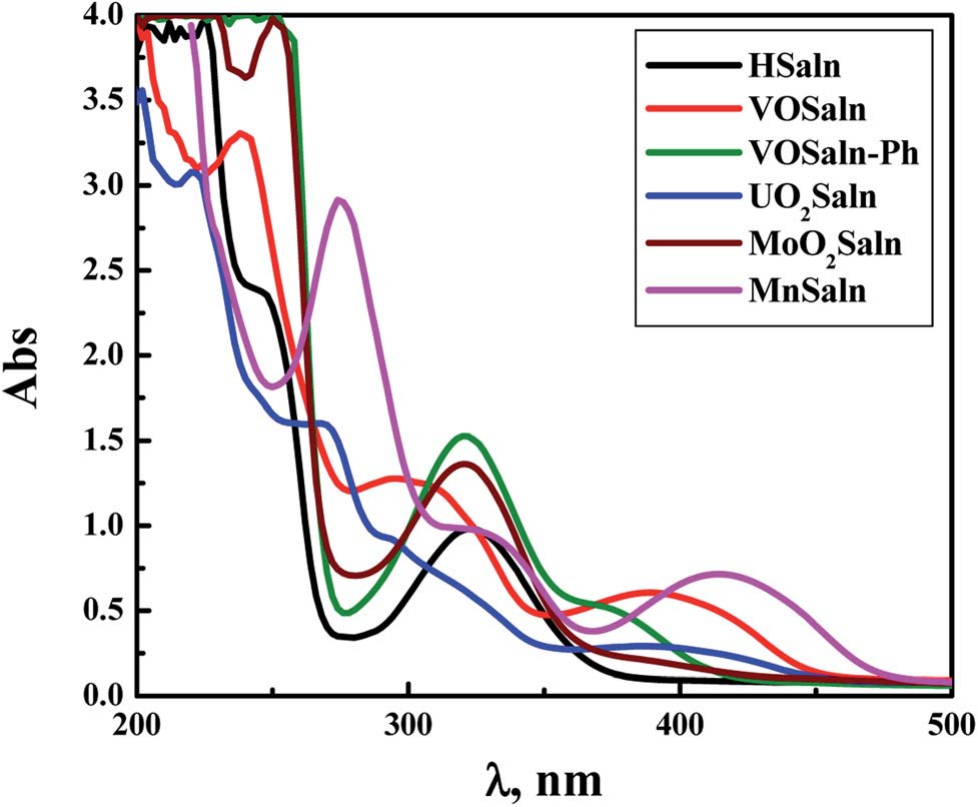
Electronic spectral scans of HSaln ligand and its MSaln complexes, [compound] = 1 × 10^−5^ mol dm^−3^ in an aqueous medium at room temperature.

The electronic transitions in the aqueous solution of all MSaln complexes were recorded at *λ*_max_, nm, with calculating the molar absorptivity, *ε*_max_, mol^−1^ cm^−1^, in Table 1 (Fig. 1). It was found some specific transitions of π → π* and n → π* in the UV region for HSaln ligand (256 and 325 nm, respectively) with little shift after bonding to the metal ions, MSaln complexes, to be 249 and 310 nm for VOSaln, 262 and 320 for MnSaln and 284 and 329 nm for MoO_2_Saln, respectively. VOSaln-Ph shows only one band at 322 nm for n → π* transition. UO_2_Saln displays two high energy bands at 238 and 271 nm for π → π* and an additional band at 299 nm for n → π* transition (Fig. 1). In the colored area of the visible region, all MSaln complexes assign broad bands at 395, 376, 388, 449 and 416 nm for probably the M-LCT transitions for VOSaln, VOSaln-Ph, UO_2_Saln, MnSaln, MoO_2_Saln, respectively.^56^ No detection for the d → d transition in any of the complexes could be observed even at higher concentration of MSaln (1.0 × 10^−3^ mmol). This result suggested that the metal complexes are in high spin-state.

The distinct infrared spectral bands values for HSaln ligand are listed in Table 2, which also affected by the presence of M^2+^ ions. The IR spectrum of all compounds is given in Fig. S10–S15, in the ESI.† From Table 2, a broad spectral band of HSaln ligand at 3401 cm^−1^, which corresponded to the hydroxy-phenolic group and/or the hydroxyl-carboxylic group, completely disappeared in all MSaln complexes. Other broad vibrating bands appeared at the same area at 3342 and 3420 cm^−1^ for VOSaln, 3135 and 3403 cm^−1^ for UO_2_Saln, 3296 and 3352 cm^−1^ for MoO_2_Saln, and 3233 and 3308 cm^−1^ for MnSaln due to the presence of the coordinated H_2_O ligands. The presence of the –OH band of VOSaln-Ph is due to the H_2_O molecule in the crystal lattice of the complex at 3368 cm^−1^. The characteristic band of Schiff base group –C=N– (1577 cm^−1^) is also highly shifted after bonding to M^2+^ ions to be 1518, 1531, 1559, 1612 and 1559 cm^−1^ for VOSaln, VOSaln-Ph, UO_2_Saln, MoO_2_Saln and MnSaln, respectively. Particularly, the single bond of C–N at 1236 cm^−1^ for HSaln ligand was also influenced by the complexation to be 1175, 1154, 1159, 1162 and 1164 cm^−1^ for VOSaln, VOSaln-Ph, UO_2_Saln, MoO_2_Saln and MnSaln, respectively. Moreover, the vibrational spectra of the C–O single bond of the phenolic and carboxylic groups was shifted due to the complexation from 1494 and 1419 cm^−1^ to be 1451 and 1422 cm^−1^ for VOSaln, 1455 and 1410 cm^−1^ for VOSaln-Ph, 1470 and 1409 cm^−1^ for UO_2_Saln, 1478 and 1403 cm^−1^ for MoO_2_Saln, and 1455 cm^−1^ for MnSaln (Table 2). The coordination feature of the carboxylate group was similar to that reported by Ziegler (2003).^55^ New stretching bands were explored in the infrared spectra of the novel M-complexes at 962, 964, 900 and 919 cm^−1^ for VOSaln, VOSaln-Ph, UO_2_Saln and MoO_2_Saln, resulted from the bond stretching of M=O. Furthermore, the coordination bonds could be found for the M–O and M–N bonding at 722 and 608 for VOSaln, 743 and 608 cm^−1^ for VOSaln-Ph, 723 and 666 cm^−1^ for UO_2_Saln, 749 and 583 cm^−1^ for MoO_2_Saln, and 751 and 601 cm^−1^ for MnSaln, respectively.^55^

Mass spectra of HSaln ligand was reported previously,^41^ whereas, that measurement for VOSaln, VOSaln-Ph, UO_2_Saln, MoO_2_Saln and MnSaln was supported by Fig. S16–S20 in the ESI.† All MSaln complexes show characteristic base peaks for the complex as [M + Na^+^] and as complex anion without sodium cation [M − Na^+^]. Evaluation of H_2_O in the M-complexes as coordinated or crystalline molecules could be established by TGA. The TGA results as mass lose percentages of the represented complexes are shown in Fig. S21 (ESI†), those all complexes have various numbers of diagnostic H_2_O molecules in the lattice or in the coordination sphere. VOSaln, MnSaln, MoO_2_Saln and UO_2_Saln complexes have mass lose percentage only in the temperature range from 180 to 230 °C (Δ*m*_exp._ = 7.9, 12.4, 3.2 and 2.5%), which are almost convenient with the calculated mass lose percentages of H_2_O in the coordination sphere (Δ*m*_cal._ = 8.1, 12.0, 3.7 and 2.8%, respectively). Hence, VOSaln, MnSaln, MoO_2_Saln and UO_2_Saln have two, three, one and one coordinated water molecules, respectively. On the other hand, VOSaln-Ph has no observed mass lose percentage in that range, however, it has mass lose percentage in the temperature range from 100 to 130 °C, which is corresponded to the crystal lattice molecules. The mass lose percentage is, Δ*m*_exp._, 3.1% referring to the presence of one H_2_O molecule in the lattice per one molecule of VOSaln-Ph complex (Δ*m*_cal._ = 2.9%, approximately).

Conductance values, *Λ*_m_, of all complexes in DMSO or DMF were measured and listed in Table 2, which informed us about the polar nature of studied compounds and the number of ions in the solution.^57^ The high conductivity of HSaln ligand and all MSaln complexes refers to that they are polar compounds with, particularly, two counter ions in their solutions, because of the presence of –SO_3_^−^ and Na^+^ ions. Magnetic features of the binary MSaln complexes proposed that VOSaln, VOSaln-Ph and MnSaln complexes have high spin *via* para-magnetic properties as 2.79, 2.24 and 5.74 B.M., respectively.

For V(iv) ion with d^1^ electronic configuration, the expected magnetism is agreed with the derived value for VOSaln and VOSaln-Ph. The observed for manganese complex with oxidation state of +2, a d^5^ ion of Mn^2+^ could exist as high spin (5 unpaired electrons) due to the magnetic moment value. The reported magnetic values in Table 2 illustrate that there is no possibility for interaction between the central metal ions in their chelates.^20^

### 2.2. Stability and thermodynamic properties

**Fig. 2:**
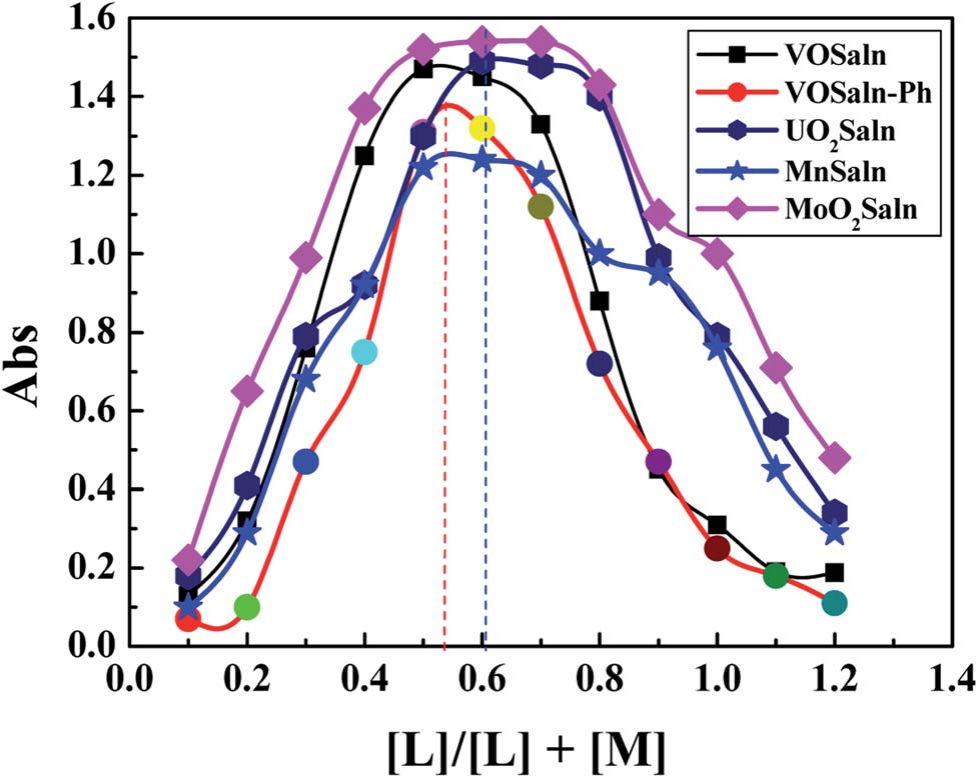
Continuous variation plots for the stoichiometric molar ratios in MSaln complexes in an aqueous- medium at [MSaln] = 1 × 10^−4^ mol dm^−3^ and 25 °C.

The stability of all MSaln complexes was determined by spectrophotometric continuous variation with appreciation of the stoichiometric molar ratios of MSaln complexes,^20,58^ which referred to that 1 equivalent of M^2+^ ion formed a complex with 1 equivalent of HSaln ligand, as presented in Fig. 2. The stability constants *K*_f_ of MSaln complexes were deduced from eqn (1)^20,41^ and tabulated in Table 3. *K*_f_ magnitudes elucidated that all MSaln complexes are quite stable in the aqueous media, moreover, UO_2_Saln is the most stable complex and VOSaln is the less stable one. The stability order could be presented for all MSaln as follow: MoO_2_Saln > UO_2_Saln > MnSaln > VOSaln-Ph > VOSaln complex.

**Table 3:** Stability constants and thermodynamic parameters of MSaln complexes [complex] = 1 × 10^−4^ mol dm^−3^

Complex	*T* (°C)	*K*_f_ × 10^8^ (L mol^−1^)	−Δ_f_*G* (kJ mol^−1^)	−Δ_f_*H* (kJ mol^−1^)	−Δ_f_*S* (J mol^−1^ K^−1^)
VOSaln	20	49.23	54.36	6.99	86.27
	25	38.31	54.67		
	30	22.93	54.29		
	35	14.45	54.00		
	40	7.58	53.20		
VOSaln	20	51.22	54.46	2.38	72.85
	25	40.99	54.83		
	30	29.08	54.89		
	35	18.12	54.58		
	40	10.50	54.05		
MoO_2_Saln	20	54.77	54.80	2.01	72.15
	25	48.75	55.03		
	30	40.12	55.13		
	35	35.65	55.33		
	40	27.49	54.19		
MnSaln	20	59.02	54.80	2.02	72.20
	25	44.41	55.03		
	30	32.01	55.13		
	35	24.26	55.33		
	40	11.08	54.19		
UO_2_Saln	20	62.18	54.93	11.50	32.52
	25	55.82	55.60		
	30	47.62	56.13		
	35	39.65	56.59		
	40	30.31	56.81		

*K*_f_ values could be applied to estimate the thermodynamic parameters values for all MSaln complexes from eqn (2) (Gibb's–Helmholtz equation as a function of the temperature reciprocal in Kelvin, 1/*T*) (Fig. S22, ESI†). The thermodynamic parameters, Δ_f_*H* and Δ_f_*S*, could be derived from eqn (2). Conclusively, the Gibb's free energy values (Δ_f_*G*) were determined as negative values. So, the spontaneous complexation between HSaln ligand and M^2+^ ion under given conditions is the result of the negative Δ_f_*G*. Moreover, the negative values of Δ_f_*H* illustrate the exothermic nature of the complexation reaction and the strong bonding between HSaln ligand and the central metal ion in all MSaln complexes.^57^

**Fig. 3:**
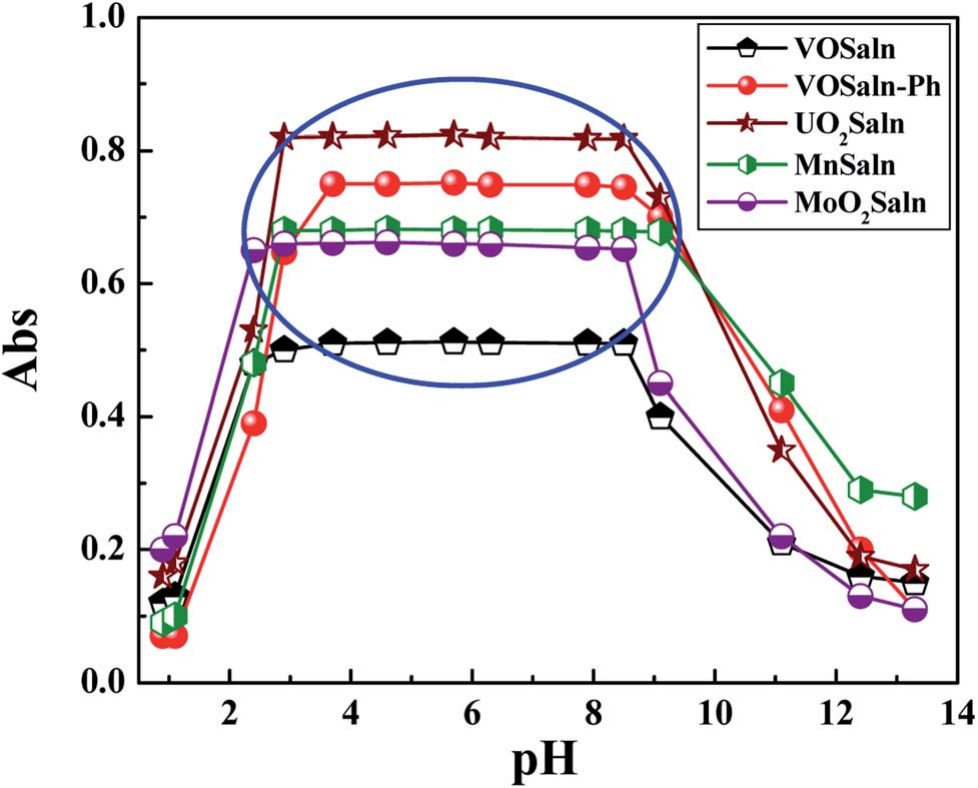
Dissociation plot of MSaln complexes in an aqueous media at various pHs.

The high stability pH range (3.1–9.7) for the new MSaln chelates and typical dissociation curves were displayed by pH-plot, *i.e.* absorbance values *versus* different pH at *λ*_max_ of each M-chelate, as demonstrated in (Fig. 3). This explained that the consistency of the metal chelate could be greatly stabilized by Schiff base ligands within complexation. Hence, proper pH extent for different applicability for the novel MSaln derives from pH = 3.1 to pH = 10.0. The proposed formula for the metal chelates is identified by correlation among the existence of magnetic measurements, molar conductance, elemental analysis, electronic spectra and infrared spectra.

### 2.3. Biological studies

#### 2.3.1. Antimicrobial potential

The results of the antimicrobial potential of the HSaln ligand and all the MSaln complexes are presented in Tables 4 and 5. As can be observed in the resulted data, there was significant antimicrobial potential of the current compounds against three different strains of bacteria and against a strain of fungi. All metal pincer chelates MSaln complexes had higher antimicrobial potential compared to their chelating agent, HSaln ligand (Tables 4 and 5).^20,24^ For the antibacterial action, the M-chelates with different central metal ions showed moderate to high potential when compared with standard antibiotic (gentamycin).^25^ The strong active Schiff base group (–CH=N–) improved the antimicrobial potential of all studied reagents, furthermore, the addition of this group increased the potential of MSaln complexes relative to the HSaln ligand. One explanation for this comes from the chelation theory effect.^59,60^ The high positive charge and the strong coordination capability of the central metal ion are considered to play an observable role in improving the antimicrobial action of the HSaln ligand. This effect was seen in the current data as the highest antimicrobial potential was seen with UO_2_Saln and MnSaln complexes. UO_2_Saln could increase its coordination number as a 4f element^61^ and also, MnSaln, which has the highest number of the labile coordinated solvent molecules.^62^ Additionally, the π-electron delocalized rings over the antimicrobial reagents and their lipophilic character could enhance their action and effect. The high solubility of the current compounds resulted from the bonded *p*-sodium sulfonate group^14^ and their conductivity could be the reason for their valuable antimicrobial action. The activity index percentages, *A*, of the antibacterial and antifungal potential of HSaln ligand and MSaln complexes, which are calculated according to eqn (3) and recorded in Tables S1 and S2 (ESI†). The antimicrobial capability of our studied compounds could also be measured by activity index percentages.

**Table 4:** Antimicrobial bioassay of HSaln ligand and MSaln complexes *versus* various strains of bacteria

Compound	Inhibition zone (mm)					
	*Serratia marcescens* (−ve)		*Escherichia coli* (−ve)		*Staphylococcus aureus* (+ve)	
Conc. (μg mL^−1^)	10	20	10	20	10	20
HSaln ligand	7 ± 0.23	10 ± 0.33	5 ± 0.21	8 ± 0.15	9 ± 0.18	14 ± 0.24
VOSaln	15 ± 0.17	34 ± 0.16	14 ± 0.43	33 ± 0.13	18 ± 0.14	40 ± 0.12
VOSaln-Ph	13 ± 0.21	30 ± 0.27	12 ± 0.24	29 ± 0.21	16 ± 0.11	36 ± 0.17
MnSaln	16 ± 0.18	36 ± 0.24	15 ± 0.14	34 ± 0.27	19 ± 0.16	42 ± 0.19
MoO_2_Saln	14 ± 0.11	33 ± 0.12	13 ± 0.04	32 ± 0.19	17 ± 0.05	38 ± 0.17
UO_2_Saln	17 ± 0.05	38 ± 0.27	16 ± 0.07	35 ± 0.19	20 ± 0.11	44 ± 0.25
Gentamycin	19 ± 0.71	40 ± 0.33	17 ± 0.15	37 ± 0.72	22 ± 0.93	46 ± 0.11

**Table 5:** Antimicrobial bioassay of HSaln ligand and MSaln complexes *versus* various strains of fungi

Compound	Inhibition zone (mm)					
	*Candida albicans*		*Aspergillus flavus*		*Trichophyton rubrum*	
Conc. (μg mL^−1^)	10	20	10	20	10	20
HSaln ligand	4 ± 0.14	7 ± 0.16	3 ± 0.21	8 ± 0.34	5 ± 0.10	11 ± 0.07
VOSaln	22 ± 0.13	34 ± 0.37	13 ± 0.18	23 ± 0.18	14 ± 0.41	29 ± 0.17
VOSaln-Ph	18 ± 0.17	29 ± 0.12	9 ± 0.17	19 ± 0.12	10 ± 0.16	25 ± 0.15
MnSaln	21 ± 0.11	33 ± 0.27	12 ± 0.27	22 ± 0.22	13 ± 0.23	28 ± 0.13
MoO_2_Saln	19 ± 0.10	32 ± 0.12	11 ± 0.10	21 ± 0.09	12 ± 0.05	27 ± 0.12
UO_2_Saln	23 ± 0.14	36 ± 0.17	14 ± 0.11	24 ± 0.21	15 ± 0.13	30 ± 0.23
Fluconazole	24 ± 0.55	37 ± 0.62	14 ± 0.71	25 ± 0.90	16 ± 0.49	31 ± 0.88

**Fig. 4:**
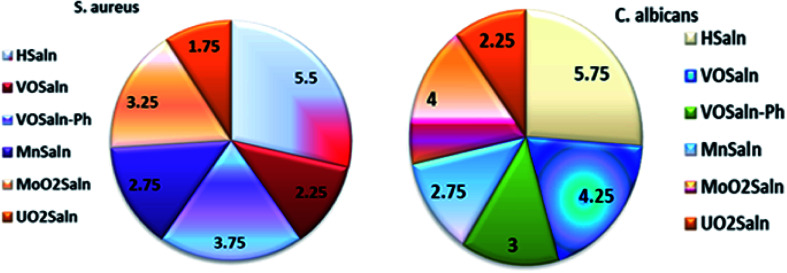
Histogram showing MIC values for antimicrobial activity of HSaln ligand and MSaln complexes.

Table 6 presents the minimum inhibitory concentration (MIC) potential of HSaln ligand and MSaln complexes *versus* both the bacteria and fungi strains. The MIC values of the HSaln ligand and its MSaln complexes *versus* the chosen bacterial and fungi strains indicate that the HSaln ligand has the lowest antimicrobial potential compared to the metal pincer chelates (Fig. 4). This suggests that the central M^2+^ ion plays an essential role in the antimicrobial potential of the studied compounds.^24^

**Table 6:** MIC, minimum inhibitory zone, for antimicrobial assay of HSaln ligand and MSaln complexes

Compound	Minimum inhibition concentration (MIC) μg mL^−1^					
	*S. Marcescens*	*E. coli*	*S. aureus*	*C. albicans*	*A. flavus*	*T. rubrum*
HSaln ligand	6.25	7.50	5.50	5.75	8.50	6.75
VOSaln	3.25	2.75	2.25	4.25	5.25	5.00
VOSaln-Ph	4.75	4.25	3.75	3.00	3.75	3.50
MnSaln	3.75	3.25	2.75	2.75	4.25	4.00
MoO_2_Saln	4.50	3.75	3.25	4.00	4.75	4.50
UO_2_Saln	2.75	2.50	1.75	2.25	3.50	3.00

The variation in the activity of the metal complexes against the different microbes possibly results from a difference in the ribosomes of the various microbial cells or a difference in the cell membrane permeability of these microbes. The lower activity of complexes as compared to others may be attributed to the low lipid solubility of these complexes. If this is the case, the metal ion may not be able to reach its site of action within the cell. Chelation itself plays a significant role in determining antibacterial behavior of the MSaln complexes but concurrently factors such as solubility, size, dipole moment, coordinating sites, redox potential of metal ion, solubility, bond length between metal and the ligand, geometry of complexes, steric, pharmacokinetic, concentration and hydrophobicity also have substantial influence on the antibacterial activity. Given that the tested complexes were more active against Gram (+ve) than Gram (−ve) bacteria, it may be concluded that the antibacterial activity of the compounds is related to the cell wall structure of the bacteria.

#### 2.3.2. CT-DNA binding

##### 2.3.2.1. Electronic spectroscopy

**Fig. 5:**
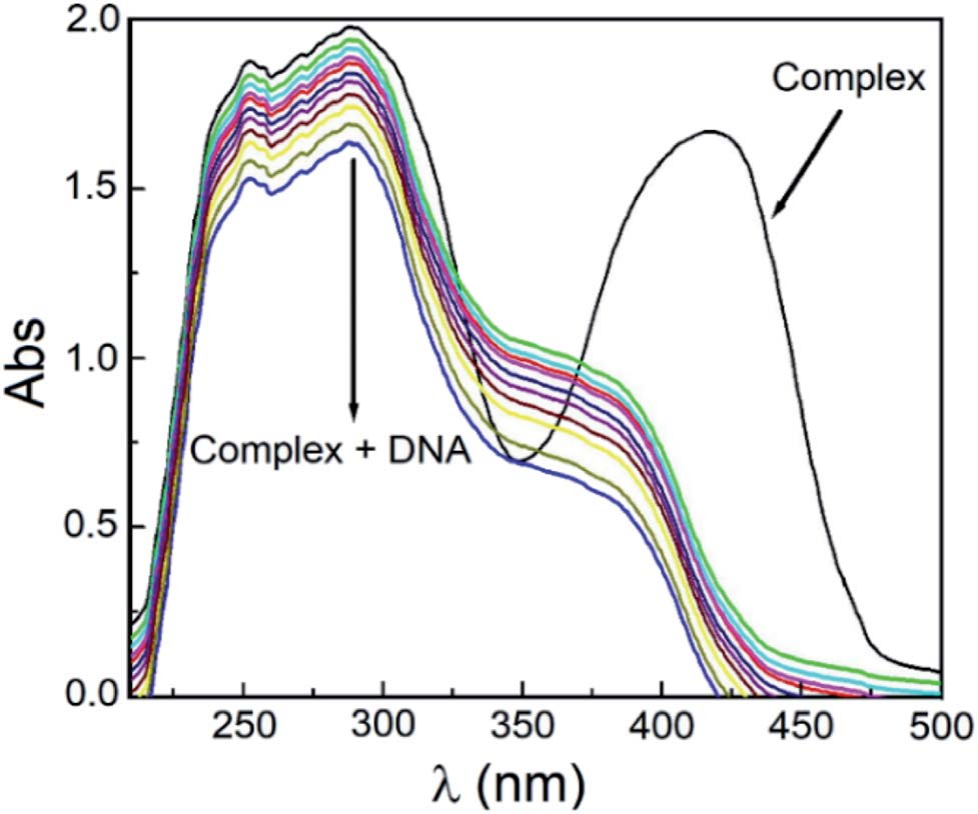
The UV-vis spectral scans of UO_2_Saln in the buffer solution in absence and presence of DNA with interval time 15 min.

**Fig. 6:**
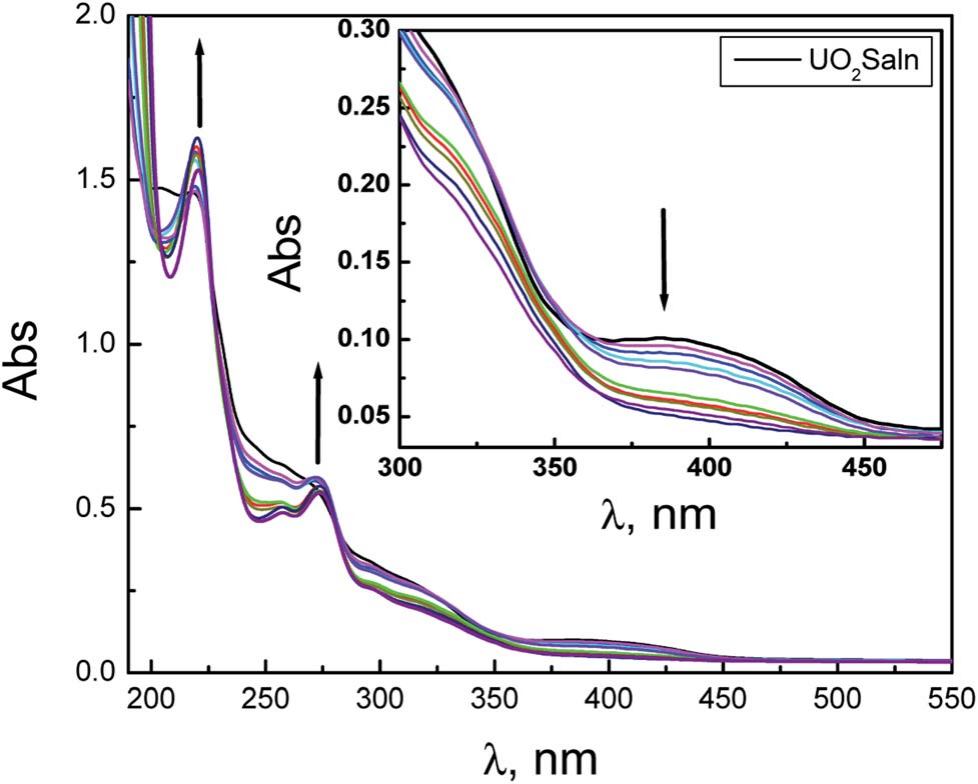
The UV-vis spectral scans of MnSaln complex in absence and presence of DNA with interval time 15 min.

The nature of bonding of MSaln complexes with DNA was estimated with electronic spectrophotometrically.^14,20,24,25^ There are three reported types of bonding with DNA; the first is an interaction of two grooved DNA double spiral, the second one is an intercalation of the arranged native DNA base pairs, and the third one is an electrostatic interaction with the nucleic sugar-phosphate negative charge (which is parallel to the external double spiral DNA without any selectivity).^20,63^ The DNA–MSaln complexes interaction was confirmed in buffered solutions of DNA by the electronic transition spectral values (Table 7), which provided the hypochromic character of that interaction and increase of the high π → π* interaction transition (Fig. 5). The nature of π → π* transition could be a result of the nitrogenous DNA base pairs and the delocalized aromatic system in the coordinated ligand in MSaln complexes.^14,64^ UO_2_Saln complex shows observable enhancement of the π → π* transition band, with increased energy due to the DNA and UO_2_Saln complex interaction (Fig. 5). Hypochromism is the consequence of interaction between the coordinated ligand orbital and the DNA base pairs orbitals *via* intercalation mode. Hence, there is a red shift in the visible region of the transition band for the interaction of DNA and MnSaln complex (from the maximum values at 449 to 368 nm; Fig. 6) with a hypochromatic character due to the decreasing of the maximum absorption.^14^

**Table 7:** UV-visible spectroscopic parameters for the DNA–MSaln complexes interaction^a^

Compound	*λ*_max_ free (nm)	*λ*_max_ bound (nm)	Δ*n*	Chromism		*K*_b_ × 10^5^	Δ*G*^≠^_b_ kJ mol^−1^
				%	Type		
MnSaln	251	240	11	4.00	Hypo	3.92	−31.20
	403	325	78	8.65			
MoO_2_Saln	252	252	0	3.80	Hypo	2.60	−30.20
	271	271	0	5.91			
	289	288	1	9.23			
	396	394	2	39.76			
VOSaln-Ph	251	252	1	2.14	Hypo	1.35	−29.27
	272	271	1	1.59			
	289	288	1	3.03			
UO_2_Saln	252	252	0	1.65	Hypo	6.50	−32.43
	320	325	5	23.93			
	386	386	0	44.62			
VOSaln	239	238	1	2.20	Hypo	1.70	−29.18
	252	251	1	2.10			
	270	269	1	2.10			
	288	286	2	2.58			
	354	352	2	7.22			
	382	376	6	19.05			

a *K*_b_, mol^−1^ dm^3^.

The measured value of the DNA–MSaln complexes interaction is the binding constant. These spectrophotometric parameters (*K*_b_) were derived from eqn (4) and are recorded in Table 7. *K*_b_ is a measure of the strength of the DNA–MSaln complexes binding interaction. The binding order of DNA with MSaln complexes is as follows: UO_2_Saln > MnSaln > VOSaln > MoO_2_Saln > VOSaln-Ph complex. From Table 7, the chromism type and values for the DNA–MSaln complexes interacting binding and Δ*n*, which points out to the characteristic absorption band shift for MSaln complexes due to its DNA-interaction. The Gibbs' free energy values Δ*G*^≠^_b_ are presented as negative values, which could be the measurement of the binding strength of DNA–MSaln complexes (Table 7). The most negative Δ*G*^≠^_b_ is for UO_2_Saln as −32.43 kJ mol^−1^ and −31.20, −30.20, −29.27 and −29.18 kJ mol^−1^ for MnSaln, MoO_2_Saln, VOSaln and VOSaln-Ph complexes, respectively. The variation in the Gibbs' free energy values between the MSaln complexes is due to the nature of the central metal ion.

Besides what is mentioned above about the nature of binding between DNA and MSaln complexes, the structural feature of MSaln complexes could have an important impact on the variation between the studied complexes in such binding. Both UO_2_Saln and MnSaln complexes have the highest interaction potential with DNA (Table 7). There are two additional factors might account for their high DNA interaction. For the UO_2_Saln complex, the central metal ion as 4f element has a higher coordination potential than transition metal ions (M^2+^) in MSaln complexes. Usually the UO_2_^2+^ ion can form complexes with more than 6 coordination number, as observed elsewhere.^55^ This could increase the strength of the bonding interaction of the UO_2_Saln complex with DNA more than the other MSaln complexes, *via* the intercalative mode (Fig. 5). Moreover, MnSaln complex has the highest number of the labile coordinated solvent molecules, *i.e.* three H_2_O,^65^ and could have progressed reactivity towards DNA binding more than the other MSaln complexes within replacement. This could be suggested by the DNA interaction reactivity of VOSaln and VOSaln-Ph complexes in which VOSaln is more reactive than VOSaln-Ph complex. The secondary coordinated ligand, liable solvent water in VOSaln and 1,10-phenanthroline in VOSaln-Ph complex, increases the DNA interaction in case of water and reduces in case of 1,10-phenanthroline. Particularly, 1,10-phenanthroline is a stronger coordinated ligand compared to water, which would increase the VOSaln-Ph complex could be bound to DNA *via* interaction more than that of the coordinated water.^27,66^ The conclusion of the Δ*G*^≠^_b_ values supports strongly the replacement mechanism.^14^

##### 2.3.2.2. Viscosity measurements

**Fig. 7:**
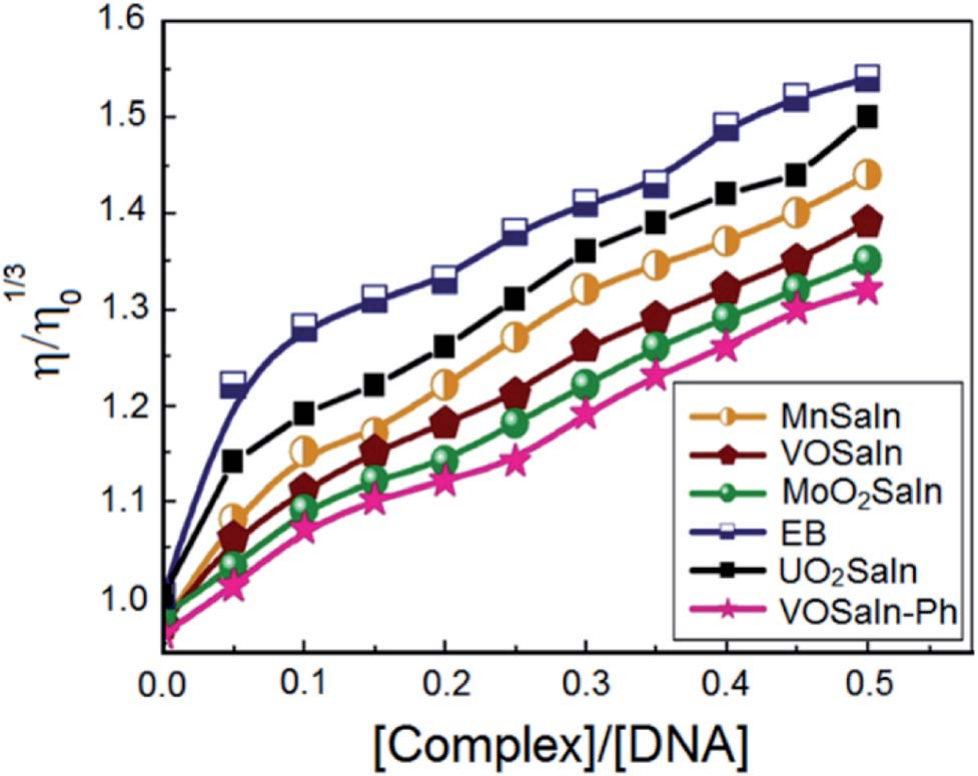
The effect of MSaln complexes amounts on the CT-DNA relative viscosity at [DNA] = 0.5 mM and 25 °C.

The measured viscosities of DNA with and without MSaln complexes can provide information on the bonding features between DNA and MSaln complexes (calculated according to eqn (7) and (8); as reported previously for different transition metal-Schiff base complexes^14,20,24^). Ethidium bromide (EB) is known to bind to DNA and cause an increase in the DNA viscosity (Fig. 7).^65^ Similarly, the interaction of DNA with increasing concentrations of MSaln complexes in the reaction media can cause increases in the DNA viscosity, *i.e.* increased DNA–MSaln complexes binding to intercalation sites in DNA.^67^

**Scheme 2:**
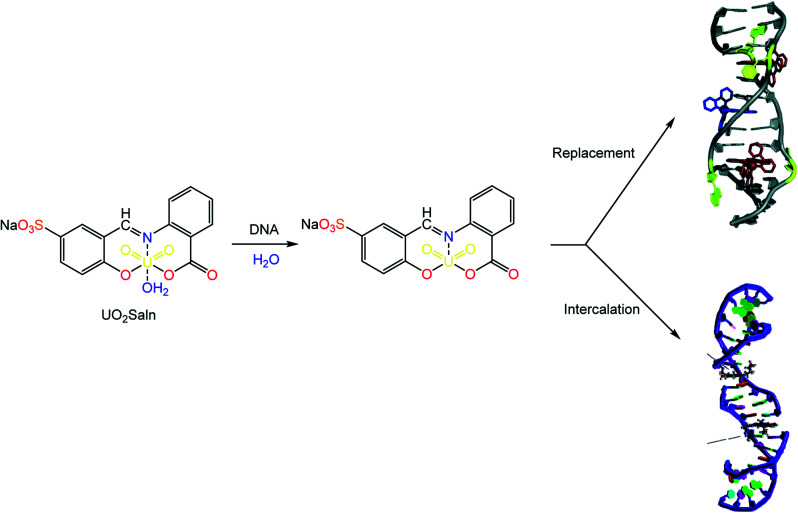
Type of interaction of DNA–UO_2_Saln complex *via* intercalation and/or coordinated water replacement bindings.

From Fig. 7, the distinction between the reacted MSaln complexes depends on the type of M^2+^ ion. UO_2_Saln complex shows the highest increase in the viscosity for a given increase in concentration. This means that UO_2_Saln complex forms the strongest complex with DNA in our studied series of MSaln complexes. The order of the viscosity measurements of the studied MSaln complexes were: UO_2_Saln > MnSaln > MoO_2_Saln > VOSaln > VOSaln-Ph complex. This indicates that the UO_2_Saln complex could be binding to DNA *via* intercalation and coordinated water replacement binding, as shown in Scheme 2.

##### 2.3.2.3. Gel electrophoresis

**Fig. 8:**
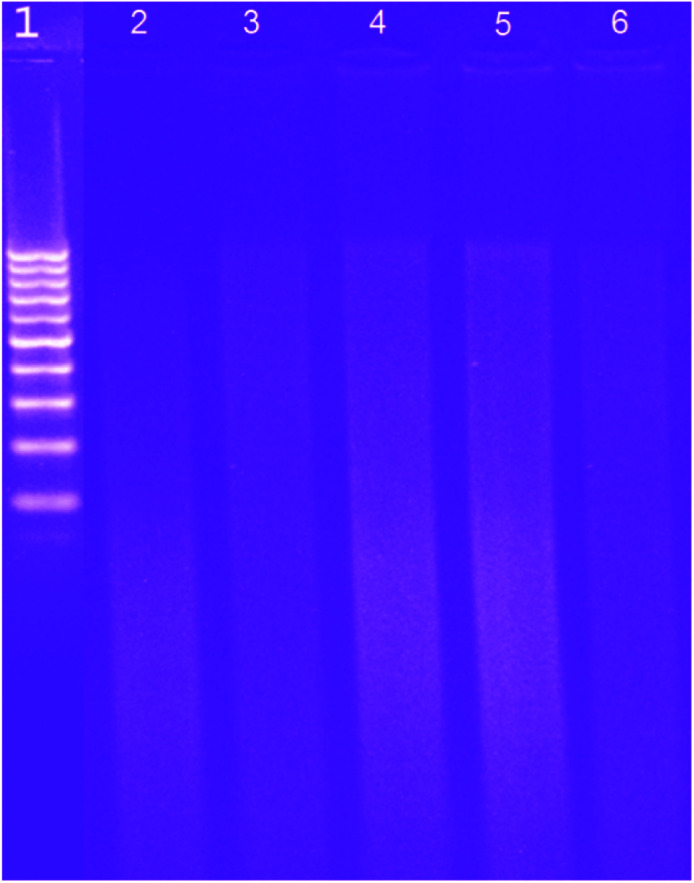
The interaction of the studied reagents with CT-DNA was studied by agarose gel electrophoresis; where lane 1: DNA ladder; lane 2: CT-DNA + VOSaln-Ph; lane 3: CT- DNA + VOSaln; lane 4: CT-DNA + UO_2_Saln; lane 5: CT-DNA + MnSaln; lane 6: CT-DNA + MoO_2_Saln.

Another important tool to probe the DNA–MSaln complexes is the gel electrophoresis.^24^ When comparing the DNA gels before and after mixing of DNA with MSaln complexes, it could be seen that the staining intensity of the gel was slightly reduced for all instances when the DNA was mixed with the MSaln complexes. With UO_2_Saln and MnSaln complexes, in particular, the DNA staining intensity disappeared, possibly due to strong DNA cleavage^14^ (Fig. 8). Therefore, it is possible that the effects of these complexes on the pathogenic organism growth could be attributed to the genomic interaction of the current MSaln complexes.

#### 2.3.3. Anticancer potential

**Fig. 9:**
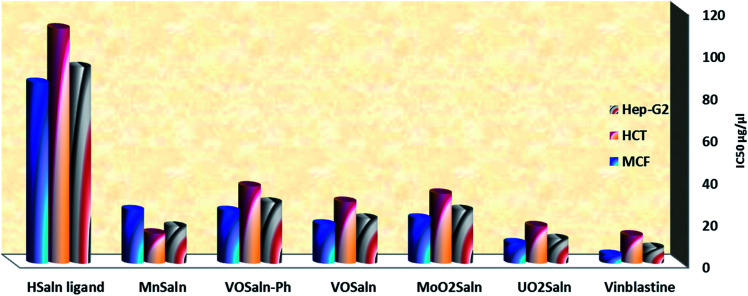
IC_50_ values of HSaln ligand and MSaln complexes against human colon carcinoma cells (HCT-116 cell line), hepatic cellular carcinoma cells (HepG-2) and breast carcinoma cells (MCF-7) cell line.

The anticancer influence of HSaln ligand and its M-complexes was examined on Hep-G2 cell line (hepatocellular carcinoma), MCF-7 cell line (breast carcinoma) and HCT-116 cell line (colon carcinoma) within concentrations ranging from 0 to 120 μg μL^−1^. The anticancer potential of the current compounds was measured by generating IC_50_ values (eqn (9)). The IC_50_ values for HSaln ligand and MSaln complexes are reported in Table S3.† The cytotoxicity data indicates that all MSaln complexes have remarkable anticancer potential, with IC_50_ values between 17.70–35.60 μg μL^−1^ for HCT-116 cell, from 9.60 to 24.90 μg μL^−1^ for Hep-G2 cell and between 11.60–28.90 μg μL^−1^ for MCF-7 cell (Fig. 9). MSaln chelates were found to be more potent anticancer reagents compared to their coordinated ligand, HSaln, and compared to the standard anticancer reagent, *i.e.* vinblastine drug. The biological effectiveness of MSaln complexes compared to HSaln ligand may be attributed to the presence of a central metal ion, the nature of which is considered in Tweed's chelation theory.^25,68^ The Lewis acid character of the metal ions (with its positive charge, especially for the high valent metals, U^6+^, Mo^6+^, V^4+^ ions) enhances the acidic character of the coordinated Schiff base ligand. Those strengths their hydrogen binding to DNA resulting in a remarkable enhancement of their biological action towards cancers.^14,24^ With all types of colon carcinoma cells, the UO_2_Saln complex has the highest biological potential as an anticancer agent. This observation is supported by all the above results involving the UO_2_Saln–DNA interaction and the antimicrobial tests. It could be concluded that the uranyl Schiff base complex is the best antimicrobial and anticancer reagent.

#### 2.3.4. Molecular docking

**Fig. 10:**
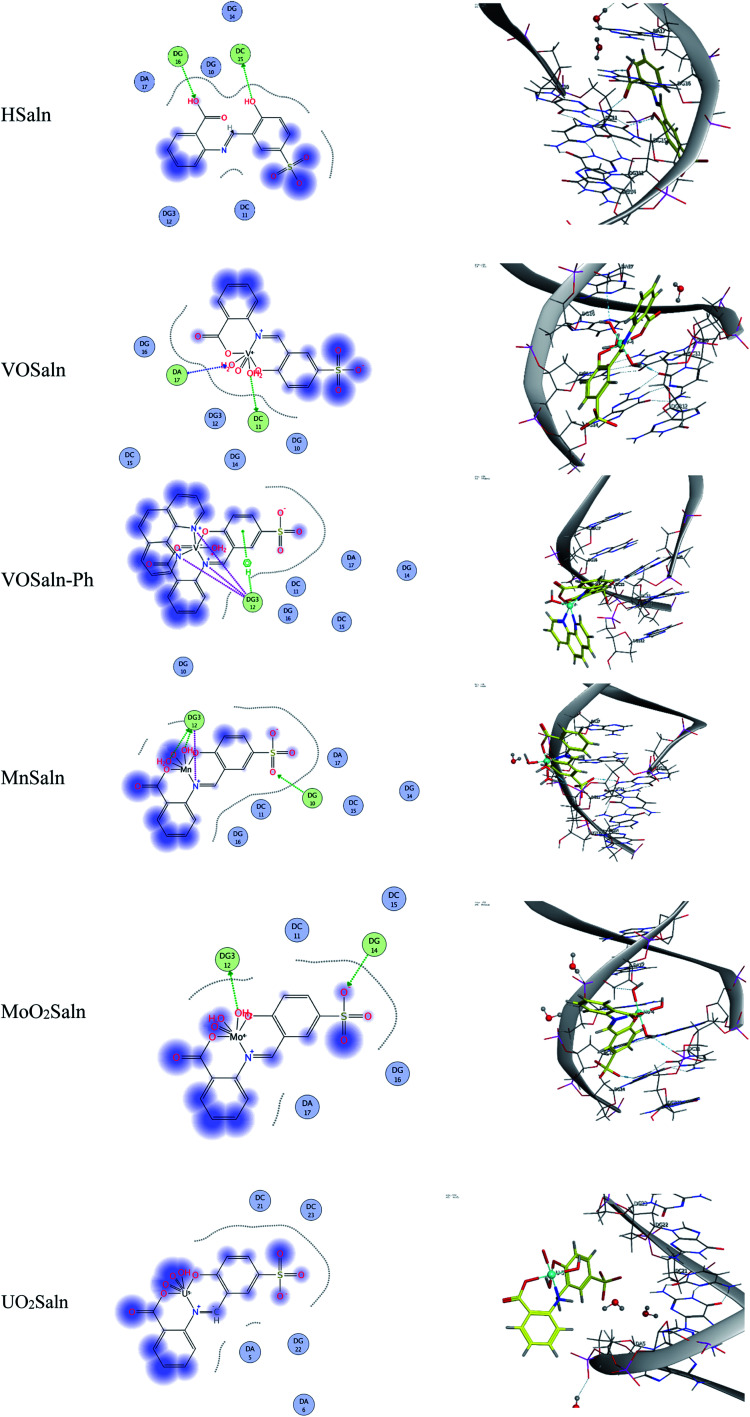
Molecular docking results of HSaln ligand and its MSaln complexes.

Molecular docking aids in understanding the nature of DNA–HSaln ligand and DNA–MSaln complex interactions,^69^ which are essential for medical proposes. The current compounds were investigated using Molecular Operating Environment (MOE) package version 2018.01 to derive the potential binding mode and energy. The binding energy (*E*_conf._) and the final score function (*S*), obtained from the MOE for the docked compounds were listed in Table 8. Molecular docking showed the hydrogen bonding interactions between HSaln ligand and B-DNA, provides the binding energy value and hydrophobic character of the interaction. The molecular docking studies showed the hydrophobic character and hydrogen bonding of HSaln ligand– and MSaln complexes–DNA, as displayed in Fig. 10. The docked highest binding score and binding energy were −5.1609 and −975.1266 kcal mol^−1^ for UO_2_Saln complex, respectively, with π-hydrogen interaction without remarking of hydrogen bonds presence with DNA.

**Table 8:** The molecular docking values for HSaln ligand and its MSaln complexes

Compound	*k*_b_	*S*	*E*_conf._
HSaln ligand	1.09	−2.2441	−100.1223
VOSaln	2.67	−4.1794	−247.6790
VOSaln-Ph	2.05	−4.0727	−206.5358
MnSaln	4.33	−4.4996	−794.3703
MoO_2_Saln	3.91	−4.3615	−551.9431
UO_2_Saln	4.76	−5.1609	−975.1266

The molecular docking studies showed that the *p*-polar substituent (*p*-sodium sulfonate group) of chelating ligand in HSaln ligand had no feasibility in binding between DNA and HSaln ligand or MSaln complexes (Fig. 10), due to their lipophobic characters. Furthermore, the functional groups of the phenolic or carboxylic hydroxyl group in HSaln ligand are seemed to be the most reactive in binding with the DNA. For the VOSaln-Ph complex, the π-hydrogen interaction could be observed due to the presence of coordinated 1,10-phenanthroline moiety, with lipophilic character.

#### 2.3.5. Catalytic potential in Suzuki–Miyaura cross-couplings

**Scheme 3:**
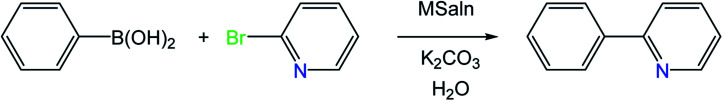
C–C cross-coupling system of phenylboronic acid and 2-bromopyridine catalyzed by MSaln complexes.

From the literatures survey, it came to our notice that the catalytic efficiency of oxy- and dioxy-high oxidation state transition metal ions, *e.g.* MoO_2_^2+^, VO^2+^ and UO_2_^2+^ ions, are not enough investigated in different cross-coupling reactions. Therefore, it gave us motivation to examine the catalytic sufficiency of MSaln chelates in standard Suzuki–Miyaura cross-coupling of phenylboronic acid with 2-bromopyridine (Scheme 3). At the optimal reaction condition, *i.e.* 6 hours heating at 100 °C, the % yield of the cross-coupling product was monitored by GC-MS which displayed in Table 9. From the obtained results, it shows that the MnSaln complex offered comparatively higher catalytic potential (provided 88% yield). All other high valent metal complexes, *i.e.* UO_2_Saln, MoO_2_Saln, VOSaln and VOSaln-Ph complexes afforded moderate to low percentages amount of the C–C product under the same sustainable conditions (50, 49, 61 and 39%, respectively), as shown in Table 9. Potassium carbonate is the most favored base for the C–C catalytic process, due to the strong interaction between the catalyst, solvent (H_2_O) and the base to enhance the catalytic potential.^70^ The solubility of organic substrates is always an issue when only water is used as a solvent in the cross-coupling reactions, though it helps to dissolve the polar inorganic reagents. As a result, it reduces the catalytic performance of the catalyst.^71,72^ Therefore most of cross coupling processes were processed in organic solvents or in mixture of organic solvent with water.^71^ Water is the most favored solvent (green solvent) for such catalytic systems, but not suitable for the solubility and miscibility of the organic substrates.^70^

**Table 9:** Suzuki–Miyaura cross coupling system of phenylboronic acid and 2-bromopyridine catalyzed by MSaln complexes^a^

Catalyst	Yield (%)^b^
No catalyst	0
VOSaln	51
VOSaln-Ph	39
MoO_2_Saln	49
MnSaln	88
UO_2_Saln	50

a Phenylboronic acid (1.0 mmol), 2-bromopyridine (1.1 mmol), base (K_2_CO_3_, 2.0 mmol) and catalyst (0.10 mmol) in 10 mL H_2_O, at 100 °C for 6 hours.

b The yield percentages of the C–C product analyzed by GC-MS.

After optimizing the reaction condition, all catalysts were tested under the same reaction condition. When the metal-complex catalyst was excluded from the reaction mixture no cross-coupling product was observed. The higher catalytic activity of the MnSaln complex could be resulted from the easily reduction of Mn^2+^ to Mn^0^ in the catalytic cycle.^73^ The higher stability of high valent metal complexes could resist the reduction step in order to prepare the active catalyst of the low oxidation state in the catalytic cycle.^38,74^ Although, Mn-complexes presented in high oxidation state Mn(v)-Salen as an effective catalyst for polymerization of furan derivatives awarding poly(silylether)s,^75^ the high valent M-complexes (UO_2_Saln, MoO_2_Saln, VOSaln and VOSaln-Ph complexes) as catalysts for such coupling systems did not promote the catalytic process. Most probably the low efficiency of UO_2_Saln, MoO_2_Saln, VOSaln and VOSaln-Ph complexes could be due to the doubly bonded oxygen to the metal ion (Mo^6+^, U^6+^ and V^4+^ ion), forming the high stable oxidation state of those metal ions, preventing its reduction to lower oxidation state in such cross-coupling mechanisms.^40,76^

As shown in Table 10, by using ionic liquids, the yield of the desired product was significantly improved in contrast to that in water catalyzed by MnSaln complex catalyst (entries 1, 3, 5 and 7, Table 9). Consequently, by using the mixture of ionic liquid with water also helped to increase the percentages yield of the C–C product (entries 2, 4, 6 and 8) due to the considered acceleration effect on such cross-couplings,^47,77^ by enhancing the solubility of the polar inorganic compounds and the substantial effect of the cations and anions on the ionic liquid, as reported elsewhere.^47,54,77^

**Table 10:** MnSaln complex catalyzed the Suzuki–Miyaura cross coupling system of phenylboronic acid and 2-bromopyridine in various ionic liquids

Entry^a^	Ionic liquid	Yield (%)^b^
1	[bmim][Tf_2_N]^c^	90
2	[bmim][Tf_2_N] : H_2_O (3 : 1)	94
3	[emim][Tf_2_N]^d^	87
4	[emim][Tf_2_N] : H_2_O (3 : 1)	91
5	[omim][Tf_2_N]^e^	84
6	[omim][Tf_2_N] : H_2_O (3 : 1)	89
7	[bmim][PF_6_]^f^	88
8	[bmim][PF_6_] : H_2_O (3 : 1)	90

a Phenylboronic acid (1.0 mmol), 2-bromopyridine (1.1 mmol), base (K_2_CO_3_, 2.0 mmol) and catalyst (0.10 mmol) in 10 mL solvent or 6.7 mL solvent : 3.3 mL H_2_O (1 : 1), at 100 °C for 6 hours.

b The yield percentages of the C–C product analyzed by GC-MS.

c 1-Butyl-3-methylimidazolium bis(trifluoromethylsulfonyl)imide.

d 1-Ethyl-3-methylimidazolium bis(trifluoromethylsulfonyl)imide.

e 1-Octyl-3-methylimidazolium bis(trifluoromethylsulfonyl)imide.

f 1-Butyl-3-methylimidazolium hexafluorophosphate.

Depending upon the nature of the substrates (electron-donating, and electron-withdrawing group on the aryl halides) the obtained product percentages yield showed variation from moderate to high, (entries 1, 2 and 3, Table 11), with aryl bromide, the yield was promoted, as seen in entries 2, 4–7 (Table 11), whereas, with the other aryl halides, *i.e.* chloride and iodide, the yield of C–C products was little reduced (entries 1, 3 and 8). When the electron-donating group on aryl bromides was attached, the yield was enhanced (entries 2, 4–6). The *o*-methyl group on bromobenzene slightly improved the percentage amount to 94% (entry 4),^47^ whereas, the *p*-methyl group or the *p*-methoxy group on bromobenzene showed slight reduction in the yield (90 or 89%) respectively.^78^ In contrast, the electronic extended conjugation or electron-withdrawing substituent (–NO_2_) on aryl halide reduced the % yield of the cross-coupling product s (entries 7 and 8).

**Table 11:** MnSaln complexes catalyzed the Suzuki–Miyaura C–C coupling reaction of phenylboronic acid and various aryl halides

Entry^a^	Aryl halide	Product	Yield (%)^b^
1	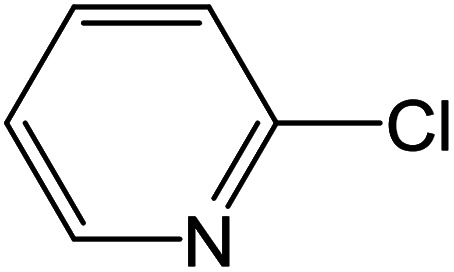	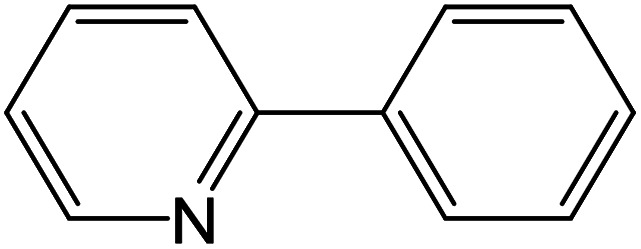	85
2	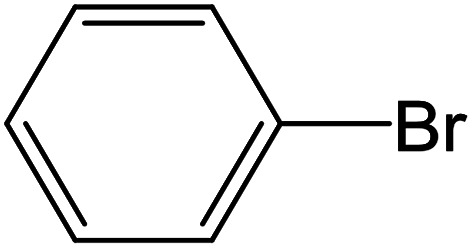	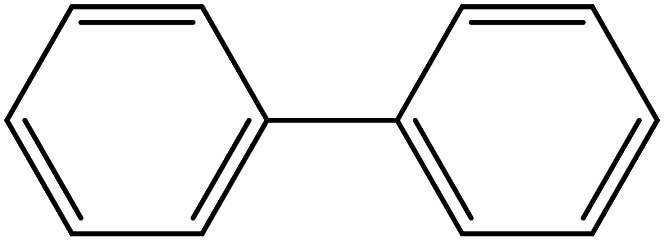	91
3	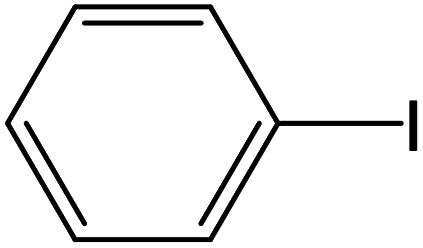	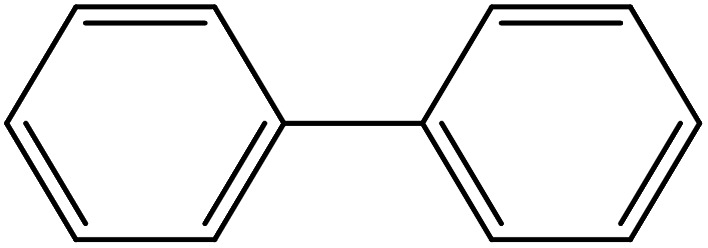	72
4	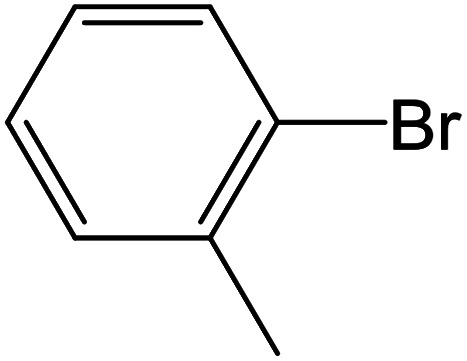	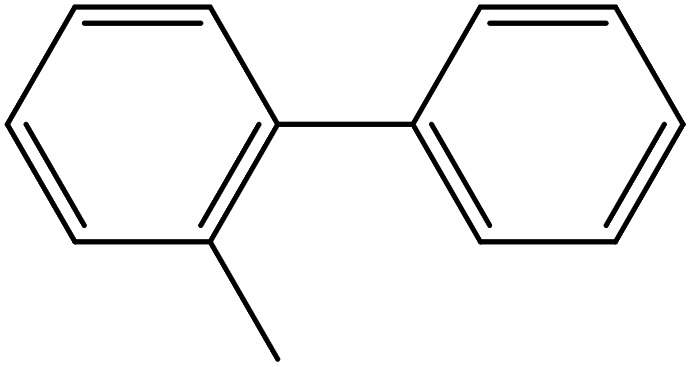	94
5	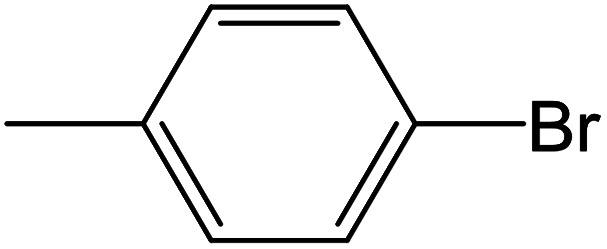	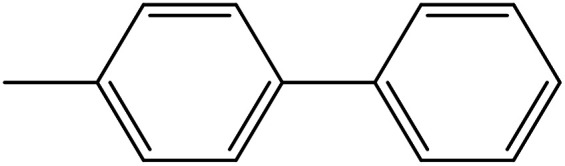	90
6	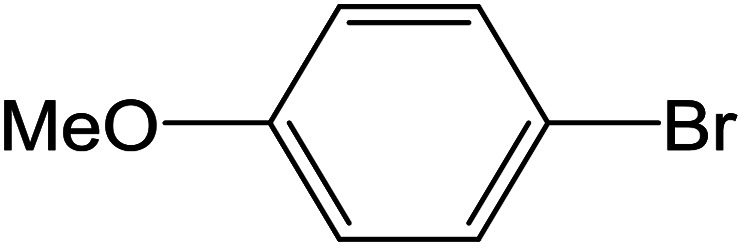	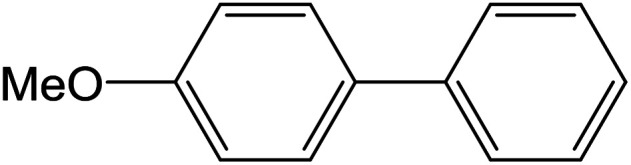	89
7	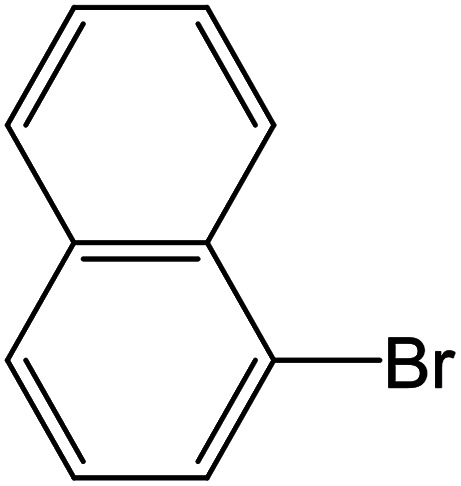	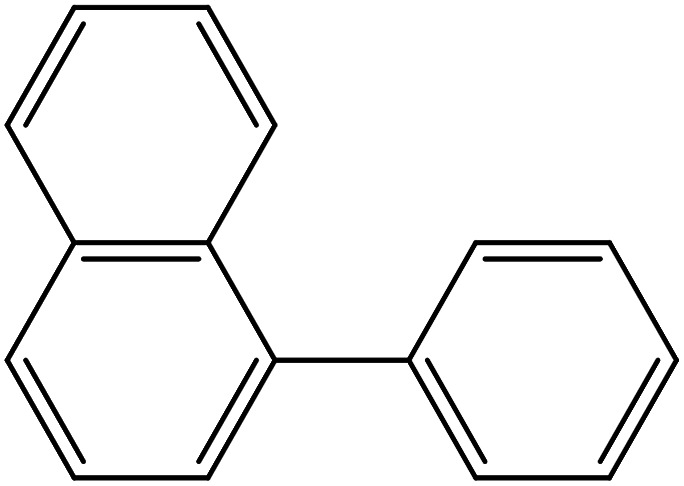	87
8	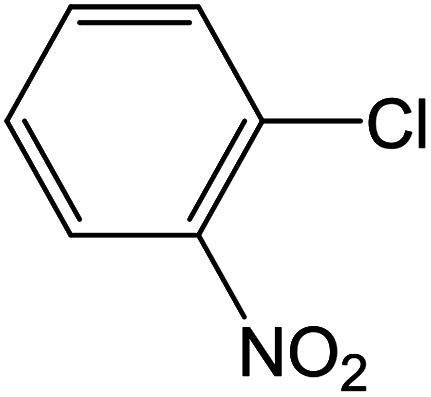	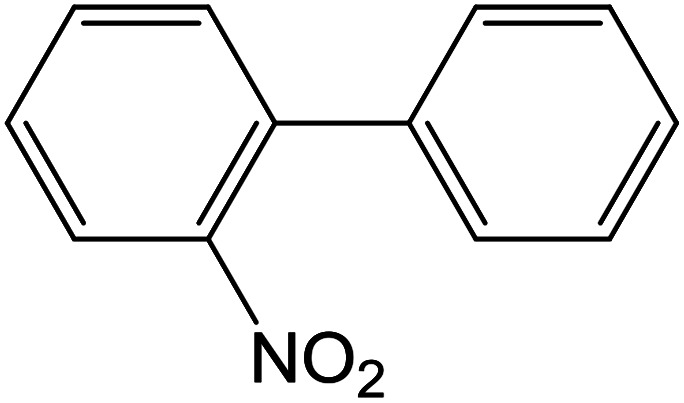	74

a Phenylboronic acid (1.0 mmol), aryl halide (1.1 mmol), base (K_2_CO_3_, 2.0 mmol) and catalyst (0.10 mmol) in 10 mL H_2_O, at 100 °C for 6 hours.

b The yield percentages of the C–C product analyzed by GC-MS.

## 3. Conclusions

The complexation of the high and low valent metal ions (VO^2+^, UO_2_^2+^, MoO_2_^2+^ and Mn^2+^ ions) with ONO-tridentate pincer Schiff base salicylidene sodium sulfonate (HSaln ligand) ligand provided water-soluble M-pincer chelates (MSaln complexes = VOSaln, VOSaln-Ph, UO_2_Saln, MoO_2_Saln and MnSaln complexes, respectively), which was fully characterized by various spectroscopic tools. Spectrophotometrically, the stability constants were derived by continuous variation method for MSaln complexes, UO_2_Saln and MoO_2_Saln are found to be the most stable complexes. Biologically, the antibacterial and antifungal potentials of MSaln complexes showed that UO_2_Saln and MoO_2_Saln complexes have the highest activity against some bacterial and fungal strains and the rest showed reasonably good potential. With selected human carcinoma cell lines, the anticancer effects of HSaln ligand and all MSaln complexes manifest high interaction. The interaction between MSaln complexes and CT-DNA with the type of binding was studied by UV-vis spectroscopy, viscosities and gel electrophoresis. All MSaln complexes exhibited good interaction with CT-DNA. The two complexes (UO_2_Saln and MnSaln complexes) demonstrated strong interaction with CT-DNA. The biological potential of MSaln complexes was studied by the molecular docking to illustrate the active site of the drug and the type of bonding between complex and DNA. As anticancer agents, all MSaln complexes and ligand showed remarkable anti-proliferative potential. The catalytic efficiency of MSaln complexes was studied in Suzuki–Miyaura cross-coupling reaction of phenylboronic acid with 2-bromopyridine in sustainable conditions. The result showed that the MnSaln complex has the highest catalytic potential, providing the highest % yield of the cross-coupling product. Moreover, the catalytic potential of the MnSaln complex was further improved in ionic liquid–aqueous mixtures (3 : 1). Generally, the biological and catalytic potential of the various MSaln complexes is following a similar trend.

## 4. Experimental

### 4.1. Reagents, apparatus and methodology

The starting materials were ordered from Acrös and Fluka, which was used as such for further work. The micro-elemental analyses of HSaln ligand and all MSaln complexes were done by a CHNS machine GMBH VarioEl model V2.3. Electronic transition spectroscopy was executed by a Jasco Ultra Violet-Visible Spectrophotometer V-570 model with 1.0 cm quartz cells in the thermostated holder at 25 °C. NMR spectroscopy was carried out by a FT-NMR multinuclear spectrometer model Bruker ARX400 at 25 °C with 400.1 MHz for ^1^H-nucleus and 100.6 MHz for ^13^C-nucleus. Mass spectroscopy was measured by the apparatus Agilent Technology 6310 (Ion Trap LC/MS – 1200 Series). Thermogravimetric analysis was obtained by using Shimadzu TGA-50H thermal analyzer under inert nitrogen atmosphere (with rate 20 cm^3^ min^−1^) as carrier gas using rate of heating 10 °C per min for the temperature range from 30 to 400 °C. IR vibrational spectroscopy was measured in the region of 4000–400 cm^−1^ as KBr pellet using Shimadzu FTIR-8101 Fourier transform infrared spectrophotometer. Conducting features were evaluated in a Jenway conductometer model 4320, supported by an epoxy bodied conductivity cell (two electrodes, shiny) with cell constant calibration from 0.01 to 19.99 at 25 °C. Magnetic measurements were achieved by a Gouy's balance with diamagnetic correction by Pascal's constants and mercury(ii) hexacyanocobaltate, as a calibrating agent, at 25 °C.

### 4.2. Synthesis

#### 4.2.1. Synthesis of HSaln ligand

HSaln ligand was prepared according to the previously reported procedure^41^ in an aqueous media using 1 equivalent of sodium salicylaldehyde-5-sulfonate with 1 equivalent of an ethanolic solution of anthranilic acid. (NMR data are presented in Fig. S1–S4 in the ESI†).

#### 4.2.2. Synthesis of VOSaln, MoO_2_Saln, MnSaln and UO_2_Saln complexes

Sustainably, an aqueous solution of vanadyl acetylacetonate, molybdenyl acetylacetonate, manganese acetate hexahydrate or uranyl acetate dihydrate (5 mmol, 30 mL) was added slowly to an aqueous solution of HSaln ligand (5 mmol, 30 mL) at room temperature. The obtained solution was heated at 70 °C for 3 hours with continuous stirring. The color of the mixed solution was changed slowly to the color of the corresponding complex. The solvent was evaporated in vacuum and the solid residue was washed with diethyl ether and dried in an oven (50 °C). The pure complexes were obtained by recrystallization with 1 : 1 mixture of methanol–water provided 69, 74, 76 and 72% yield of VOSaln, MoO_2_Saln, MnSaln and UO_2_Saln complexes, respectively.

#### 4.2.3. NMR spectra of MoO_2_Saln and UO_2_Saln complexes

MoO_2_Saln, ^1^H NMR (DMSO-*d*_6_, 400 MHz): *δ* 3.35 (s, 2H, H_2_O overlapped with water of DMSO-*d*_6_), 6.94 (dd, ^3^*J* = 8.1 Hz, 2H), 7.34 (t, ^3^*J* = 7.9 Hz, 1H), 7.55 (d, ^3^*J* = 7.9 Hz, 1H), 8.54 (d, ^4^*J* = 2.1 Hz, 1H), 8.82 (s, 1H), 10.53 (s, 1H) and 11.00 ppm (s, 1H, CH=N) (Fig. S5 and S6†).

^13^C NMR (100 MHz, DMSO-*d*_6_, Dept-135): *δ* 116.55 (CH), 116.83 (CH), 119.82 (CH), 120.07 (C_q_), 126.86 (CH), 129.74 (CH), 131.53 (C_q_), 141.06 (C_q_), 147.07 (CH), 156.74 (CH), 157.73 (C_q_), 162.84 (C_q_), 163.41 (CH, CH=N) and 169.92 ppm (C_q_, C=O).

UO_2_Saln, ^1^H NMR (DMSO-*d*_6_, 400 MHz): *δ* 2.43 (s, 2H, H_2_O), 6.98 (d, ^3^*J* = 8.0 Hz, 1H), 7.20 (d, ^3^*J* = 8.0 Hz, 1H), 7.40 (t, ^3^*J* = 8.0 Hz, 1H), 7.59 (t, ^3^*J* = 8.0 Hz, 1H), 7.81 (d, ^3^*J* = 8.0 Hz, 1H), 8.02 (s, 1H), 8.19 (d, ^3^*J* = 8.0 Hz, 1H) and 9.34 ppm (s, 1H, CH=N) (Fig. S7–S9†).

^13^C NMR (100 MHz, DMSO-*d*_6_, Dept-135): *δ* 110.23 (CH), 117.03 (CH), 121.52 (CH), 126.95 (CH), 131.55 (C_q_), 132.73 (C_q_), 134.25 (CH), 151.57 (C_q_), 155.57 (C_q_), 161.20 (CH, CH=N) and 174.11 ppm (C_q_, C=O).

#### 4.2.4. Synthesis of VOSaln-Ph complex

To an aqueous solution of VO-Saln (5 mmol, 30 mL), a methanolic solution of 1,10-phenanthroline (5 mmol, 10 mL) was added at room temperature. The mixture was heated at 70 °C for 3 hours with continues stirring. The color was changed slowly from dark green to brownish-green. The solvent was evaporated in vacuum and the solid residue was washed with diethyl ether and dried in an oven (50 °C). The pure complexes were obtained by recrystallization with 1 : 1 mixture of methanol–water.

### 4.3. Stability constants of the prepared MSaln complexes

All MSaln complexes were synthesized by the reaction of equimolar ratios of the metal ion and HSaln ligand. This could be determined by the continuous variation method,^20^ which is helpful to deduce the MSaln complexes stability constants *K*_f_ at various applied temperatures (20, 25, 30, 35 and 40 °C),^41^ by using eqn (1):1
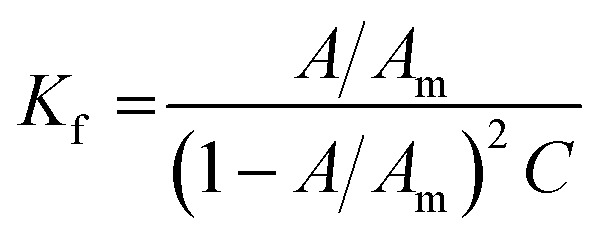


The Gibb's free energy (Δ_f_*G*) of all MSaln complexes was calculated according to eqn (2), and the thermodynamic parameters, Δ_f_*H* and Δ_f_*S*, were deduced from the Gibb's–Helmholtz equation as function reciprocal of *T* in kelvin:

2 Δ_f_*G* = −*RT* ln *K*_f_

where, *K*_f_ is the stability constant, *R* represents the gas constant and *T* (K) represents the temperature.

### 4.4. Antibacterial investigations

HSaln ligand and its corresponding M-chelates were examined as antibacterial reagents. Using DMSO (dimethylsulfoxide) as a solvent and the agar well dilution method,^14^ three different bacterial strains, *Serratia marcescens* (−ve), *Escherichia coli* (−ve) and *Staphylococcus aureus* (+ve) using were evaluated. The various bacterial strains were grown on nutrient agar in sterile Petri plates at room temperature. In holes created by using a sterile cork borer, a sterile paper saturated with a solution (10 and 20 μg mL^−1^) of HSaln ligand or its MSaln complexes was placed. The resultant plates were incubated for 1 day at 37 °C.^14^ Gentamycin, was used as a positive control. There was no antibacterial potential of the DMSO alone.

### 4.5. Antifungal investigations

By applying the well diffusion method, HSaln ligand and its MSaln complexes were studied as antifungal reagents *versus* three fungal strains, *Candida albicans*, *Aspergillus flavus* and *Trichophyton rubrum*, under the environment of potato dextrose agar.^24^ From the infected areas of the host plant, the applied fungal species were extracted, mixed with potato dextrose agar and poured in Petri dishes. Using DMSO as a solvent, the compounds (10 and 20 μg mL^−1^) were applied to the Petri dishes. The resultant plates were then incubated for 72 hours at 35 °C. Fluconazole was used as a positive control.

### 4.6. Minimal inhibitory concentration (MIC) and activity index (A), of antimicrobial potential

To determine the MIC, a range of compound concentrations were tested. The zone of inhibition (in mm) of the investigated metal complexes was compared with available standard drugs. The MIC is defined as the smallest concentration of the compound that inhibits the growth of the microorganisms.

The activity index (A), is calculated from eqn (3):^24,25^3
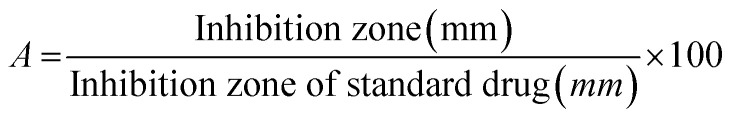


### 4.7. Interaction of CT-DNA with MSaln chelates

A stock solution of CT-DNA was prepared by mixing Tris–HCl (5 mM) and NaCl (50 mM) in deionized water (pH = 7.5). The UV absorbance of the DNA stock solution was measured at 260 nm and its molar absorption coefficient calculated (6600 mol^−1^ cm^−1^). The electronic absorbance ratio (*A*_260_/*A*_280_) of the stock DNA solution was 1.83 (almost between 1.8–1.9) indicating that there was no protein contamination of the prepared CT-DNA solution.^25^ The DNA stock solution was stored at 4 °C and used for 4 days only. The ethidium bromide (EB) concentration was evaluated at 480 nm (*ε* = 5860 mol^−1^ cm^−1^). The interaction-binding experiments were performed with 10^−3^ M Tris-buffer (Tris–HCl (5 mM) and NaCl (50 mM) in Tris-buffer with pH = 7.5). Solutions of MSaln complexes were prepared in fresh DMSO (VOSaln, VOSaln-Ph, UO_2_Saln, MnSaln and MoO_2_Saln).

#### 4.7.1. UV-visible spectroscopy

Using a given fixed molar concentration of MSaln complexes in DMSO, the electronic spectra were examined in the presence of various CT-DNA molar concentrations. The absorbance values of CT-DNA alone (in absence of MSaln complex) were removed by adding the same concentration to the blank compartment. In this manner, the spectra changes could be attributed to the DNA–MSaln complexes. The binding constant, *K*_b_, was derived by plotting [DNA]/(*ε*_a_ − *ε*_b_) against the concentrations of DNA, [DNA] from eqn (4):^14,24^4
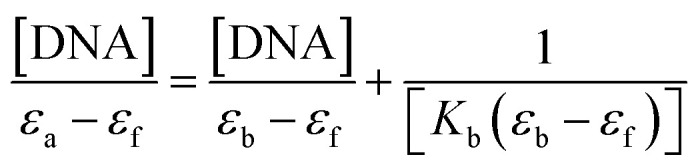
where, [DNA] is the CT-DNA concentration in mmoles, the extinction coefficients of free, apparent and complete binding of DNA with the reacting compound (MSaln complexes) are represented as *ε*_f_, *ε*_a_ and *ε*_b_, respectively. From the isolated compound (MSaln complexes) calibration curve of *A*_abs_/[complex], as well as, CT-DNA calibration curve of *A*_abs_/[DNA], the values of *ε*_f_ and *ε*_a_ were calculated, respectively. *K*_b_ was calculated from the slope and the intercept ratio of the above plotting. The standard Gibb's free energy, Δ*G*^≠^_b_, for the interacted CT-DNA with MSaln complexes, was recorded from eqn (5):^24^

5 Δ*G*^≠^_b_ = *RT* ln *K*_b_

The type and magnitude of the chromism could be established based on eqn (6):6
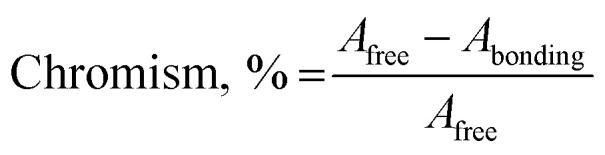
where, *A*_free_ is the absorbance of MSaln complexes in the absence of DNA and *A*_bonding_ is the absorbance at the maximum absorption band of MSaln complexes with DNA in different concentrations.

#### 4.7.2. Hydrodynamic measurements

The hydrodynamic measurements of the studied metal complexes (MSaln complexes) with CT-DNA mixtures at 25 °C were estimated as relative viscosities by using Ostwald micro-viscometer. The time consumed for fluidity (in seconds) of different concentrations of MSaln complexes, in the absence and to various concentrations of MSaln complexes from 5.0 to 50.0 μM under inert atmosphere, *i.e.* bubbling of nitrogen gas through the mixed solution in the viscometer. By applying eqn (7), the viscosity was determined in the absence (*η*°) and presence of different concentrations of MSaln complexes with CT-DNA (*η*):^24,25^7
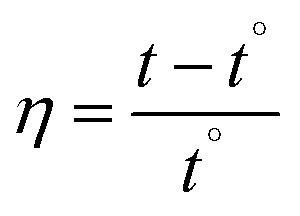
where, *t* is the fluid time of the mixed MSaln complexes with CT-DNA (seconds) and *t*° is the buffer fluid time. The relative viscosity, *η*/*η*°, was derived from the plotting the viscosity *versus* 1/*R*, where *R* was confirmed from eqn (8):8
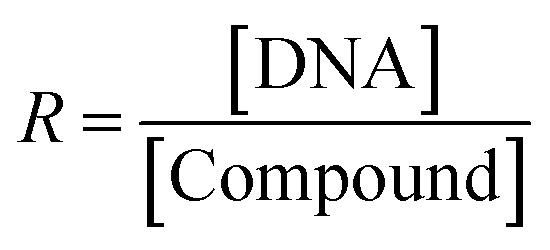
[DNA] is the CT-DNA concentration and [compound] is the concentration of MSaln complexes.

#### 4.7.3. Agarose gel electrophoresis

One of the most favored tools to evaluate the binding of the studied compound with DNA is agarose gel electrophoresis.^14,24^ A stock solution of MSaln complexes was prepared by dissolving 20.0 mg of MSaln complexes in 20 mL of DMF. 25.0 μg mL^−1^ of MSaln complexes was mixed with separated DNA of CT-DNA (calf-thymus) and incubated at 37 °C for 1 hour. Then, 30.0 μL was taken from the resulted interacted MSaln complexes–DNA solution and mixed with bromophenol blue dye with equimolar ratios (1 : 1) and then charged to the electrophoresis chamber wells parallel to a sample of the standard DNA marker in TBE buffer (50 mM Tris-base in pH 7.2 with 1 mM EDTA/1.0 l). The gel was run using 60 V for approximately 45 min.

The agarose gel was prepared by dissolving 600.0 mg of agarose in 60.0 mL of hot Tris-acetate-EDTA buffer (TAE) (4.84 g Tris-base, pH 8.0 and 0.5 M EDTA L^−1^). The obtained solution was boiled for a few minutes. After cooling, the solution was poured into a fitted gas cassette with the comb. The gel was allowed to cool gradually to room temperature until to be solid. The solid gel was placed in the electrophoresis chamber containing TAE buffer. The agarose gel was imagined and photographed in genius 3 Panasonic DMC-LZ5 Lumix DNA gel documentation system.

### 4.8. Anticancer potential

The anticancer potential of HSaln ligand and its MSaln complexes was examined at Cancer Biology Department, Pharmacology Department, Cairo University and the National Cancer Institute. Anti-cancer potential was tested against a HepG2 cell line (hepatocellular carcinoma), a MCF7 cell line (breast carcinoma) and an HCT-116 cell line (colon carcinoma), spectroscopically, the absorbance of each current compound was determined with an ELISA microplate reader (Meter tech. Σ 960, “USA”) at 564 nm. The anticancer potential was visualized *in vitro* using the Sulfo-Rhodamine-B-stain (SRB).^25^ To this end, a solution of HSaln ligand or MSaln complexes in DMSO (various concentrations 0, 1.0, 2.5, 5.0 and 10.0 μg μL^−1^) was poured dropwisely into a 96-multiwell plate, that contained a mono-layer of the cancer cells (104 cells per well). The plate was then incubated at 37 °C for 48 h under inert atmosphere carbon dioxide (5%). After the addition of Sulfo-Rhodamine-B-stain (SRB), the plate was fixed. Excess stain was removed by acetic acid and then mixed with Tris–EDTA buffer. An ELISA plate reader was used to determine the color intensity. The inhibitory concentration (IC_50_) was deduced by applying eqn (9):^25^9
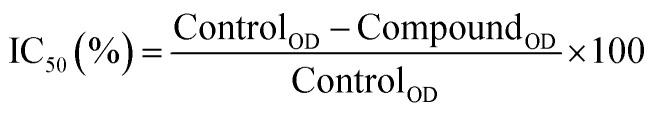


### 4.9. Molecular docking

Dell Precision™ T3600 Workstation Intel Xeon E5-1660 3.3 GHz, 16 GB 1600 MHz DDR3, ECC RDIMM 1 TB (7200 RPM), 1 GB NVIDIA Quadro 2000, Windows 10 Professional (64 Bit) was applied in order to investigate the current compounds molecular docking (HSaln ligand and all MSaln complexes) with using Molecular Operating Environment (MOE) package version 2018.01. X-ray analysis of a B-DNA dodecameric d(CGCG AATTCGCG)2 crystal structure within 3′–5′direction (DNA code: 1BNA) was applied in order to examine the docking at a resolution of 1.9 Å. By importing the molecular structure of DNA with its H atoms into MOE, the optimization energy could be calculated. The obtained structural model was submitted to the systematic conformation search within a gradient of RMS of 0.01 kcal mol^−1^ with negligible parameters in the Site Finder tool embedded in MOE. By means of ChemBio Draw Ultra 12.0, all HSaln ligand and its MSaln complexes were drawn. For calculating the parameters of the investigated compounds using molecular docking following changes were done: (1) adding the hydrogen atoms; (2) applying the conformational search for all HSaln ligand and its MSaln complexes; (3) MMFF94 force field, the best conformers obtained showed an energy decrease.^79^ Energy reduction with the steepest algorithm was exercised, after which the conjugate gradient method was applied until an RMS gradient reached 0.00001 kcal mol^−1^ Å^−1^. In MOE 2016.08, the standard molecular docking was exercised. The Alpha Triangle placement that derives poses by random superposition of HSaln ligand atoms triplets alpha sphere dummies in the target site was used to detect the poses. The London dG scoring function elucidated the free binding energy of the ligand from a given pose. The best poses of the docked molecules were estimated by at least 50 cycles of calculation. The obtained molecular dock file was created with different poses for HSaln ligand and its MSaln complexes with rearrangement with the final score function (*S*). The score of the last stage (*S*) is that was not set to none. To investigate the different poses, the browser of resulted database was selected for the best.

### 4.10. Catalytic processes

Suzuki–Miyaura cross-coupling processes were carried out in two necked round bottom flask (100 mL) with water circulatory glass condenser, loaded with phenylboronic acid (1.0 mmol) in 10 mL H_2_O, 2.1 mmol of potassium carbonate and charged with 0.10 mmol of MSaln complexes catalyst (VOSaln, VOSaln-Ph, MnSaln, MoO_2_Saln or UO_2_Saln). The reaction was initiated by the addition of 1.2 mmol of 2-bromopyridine and the reaction mixture was heated under reflux conditions at 110 °C for 6 hours in a thermostated oil bath.

The cross-coupling product was extracted by diethyl ether (30 mL) and dried by anhydrous magnesium sulfate and further diluted with 10 mL of diethyl ether to make a dilute sample for GC-MS analysis. The yield (in percentages) of C–C products were resolved with Shimadzu Gas Chromatography Mass Spectrometer (GC-MS) model QP2010 SE, with Rxi-5 Sil MS capillary column (30 m length × 0.25 mm ID × 025 μm film thickness). The sample was injected into the GC-MS system at 25 °C. The GC oven temperature was initiated at 40 °C and fixed for 60 seconds. The rate of the temperature with 10 °C min^−1^ was increased up to 200 °C. The operation of the inlet was carried out in the mode of splitless at 200 °C, which was the mass spectra temperature of the transfer line. The carrier gas was pure helium (99.999%), which flowed with rate of 1 mL min^−1^. Lab solution software was applied to analyze the yield percentages of the obtained products.

## Conflicts of interest

There is no conflict of interest in this manuscript.

## Supplementary Material

RA-009-C9RA06816C-s001

## References

[cit1] Vigato P. A., Tamburini S. (2004). Coord. Chem. Rev..

[cit2] Clarke R. M., Storr T. (2014). Dalton Trans..

[cit3] Shoair A. F., El-Shobaky A. R., Abo-Yassin H. R. (2013). J. Mol. Liq..

[cit4] Abdel-Rahman L. H., Abu-Dief A. M., Mostafa H., Hamdan S. K. (2017). Appl. Organomet. Chem..

[cit5] Abdel-Rahman L. H., Abu-Dief A. M., Shehata M. R., Atlam F. M., Abdel-Mawgoud A. A. H. (2019). Appl. Organomet. Chem..

[cit6] Lehwess-Litzmann A., Neumann P., Parthier C., Lüdtke S., Golbik R., Ficner R., Tittmann K. (2011). Nat. Chem. Biol..

[cit7] Liu X., Manzur C., Novoa N., Celedón S., Carrillo D., Hamon J.-R. (2018). Coord. Chem. Rev..

[cit8] Adaeo P., Kuznetsov M. L., Barroso S., Martins A. M., Avecilla F., Pessoa J. C. (2012). Inorg. Chem..

[cit9] Gong D., Wang B., Jia X., Zhang X. (2014). Dalton Trans..

[cit10] Das P., Linert W. (2016). Coord. Chem. Rev..

[cit11] Beigi Z., Kianfar A. H., Farrokhpour H., Roushani M., Azarian M. H., Mahmood W. A. K. (2018). J. Mol. Liq..

[cit12] Al Zoubi W., Al Mohanna N. (2014). Spectrochim. Acta, Part A.

[cit13] Jeevadason A. W., Murugavel K. K., Neelakantan M. A. (2014). Renewable Sustainable Energy Rev..

[cit14] Adam M. S. S., Elsawy H. (2018). J. Photochem. Photobiol., B.

[cit15] Correia I., Roy S., Matos C. P., Borovic S., Butenko N., Cavaco I., Marques F., Lorenzo J., Rodríguez A., Morenoe V., Pessoa J. C. (2015). J. Inorg. Biochem..

[cit16] Krivosudský L., Schwendt P., Gyepes R., Zák Z. (2014). Polyhedron.

[cit17] Krivosudský L., Schwendt P., Šimunek J., Gyepes R. (2015). J. Inorg. Biochem..

[cit18] Pessoa J. C. (2015). J. Inorg. Biochem..

[cit19] Lu L., Yue J., Yuan C., Zhu M., Han H., Liu Z., Guo M. (2011). J. Inorg. Biochem..

[cit20] Adam M. S. S., Youssef M. M., Abo Elghar M. F., Hafez A. M., El-Ayaan U. (2017). Appl. Organomet. Chem..

[cit21] Kazemi Z., Rudbari H. A., Sahihi M., Mirkhani V., Moghadam M., Tangestaninejad S., Mohammadpoor-Baltork I., Gharaghani S. (2016). J. Photochem. Photobiol., B.

[cit22] Rauf A., Shah A., Khan A. A., Shah A. H., Abbasi R., Qureshi I. Z., Ali S. (2017). Spectrochim. Acta, Part A.

[cit23] Saini A. K., Kumari P., Sharma V., Mathur P., Mobin S. M. (2016). Dalton Trans..

[cit24] Abdel-Rahman L. H., El-Khatib R. M., Nassr L. A. E., Abu-Dief A. M., Ismael M. (2014). Spectrochim. Acta, Part A.

[cit25] Abdel-Rahman L. H., Abu-Dief A. M., El-Khatib R. M., Abdel-Fatah S. M. (2016). Bioorg. Chem..

[cit26] Shahzadi S., Ali S., Sharma S. K., Qanungo K. (2010). J. Iran. Chem. Soc..

[cit27] Abd El-Halim H. F., Mohamed G. G., Khalil E. A. M. (2017). J. Mol. Struct..

[cit28] Liu Y.-T., Sheng J., Yin D.-W., Xin H., Yang X.-M., Qiao Q.-Y., Yang Z.-J. (2018). J. Organomet. Chem..

[cit29] Sharaby C. M., Amine M. F., Hamed A. A. (2017). J. Mol. Struct..

[cit30] Mahmoud W. H., Mahmoud N. F., Mohamed G. G. (2017). Appl. Organomet. Chem..

[cit31] Mahmoud W. H., Deghadi R. G., El Desssouky M. M. I., Mohamed G. G. (2018). Appl. Organomet. Chem..

[cit32] Mahmoud W. H., Omar M. M., Sayed F. N., Mohamed G. G. (2018). Appl. Organomet. Chem..

[cit33] Mahmoud W. H., Deghadi R. G., Mohamed G. G. (2016). Res. Chem. Intermed..

[cit34] Rasool R., Hasnain S. (2015). Spectrochim. Acta, Part A.

[cit35] Belal A. A. M., El-Deen I. M., Farid N. Y., Zakaria R., Refat M. S. (2015). Spectrochim. Acta, Part A.

[cit36] Zayed E. M., Hindy A. M. M., Mohamed G. G. (2018). Appl. Organomet. Chem..

[cit37] Zayed E. M., Hindy A. M. M., Mohamed G. G. (2018). Appl. Organomet. Chem..

[cit38] Zayed E. M., Mohamed G. G., Hassan W. M. I., Elkholy A. K., Moustafa H. (2018). Appl. Organomet. Chem..

[cit39] Zayed E. M., Zayed M. A., Hindy A. M. M., Mohamed G. G. (2018). Appl. Organomet. Chem..

[cit40] Han F.-S. (2013). Chem. Soc. Rev..

[cit41] Abd El-Lateef H. M., Adam M. S. S., Khalaf M. M. (2018). J. Taiwan Inst. Chem. Eng..

[cit42] Adam M. S. S., Mohamad A. D. M. (2018). Polyhedron.

[cit43] Scalese G., Benítez J., Rostán S., Correia I., Bradforda L., Vieites M., Minini L., Merlino A., Coitiño E. L., Birriel E., Varela J., Cerecetto H., González M., Pessoa J. C., Gambino D. (2015). J. Inorg. Biochem..

[cit44] Rull S. G., Funes-Ardoiz I., Maya C., Maseras F., Fructos M. R., Belderrain T. R., Nicasio M. C. (2018). ACS Catal..

[cit45] Suganthy P. K., Prabhu R. N., Sridevi V. S. (2016). Inorg. Chim. Acta.

[cit46] Abdel Aziz A. A., Salem A. N. M., Sayed M. A., Aboaly M. M. (2012). J. Mol. Struct..

[cit47] Wang M., Yuan X., Li H., Ren L., Sun Z., Hou Y., Chu W. (2015). Catal. Commun..

[cit48] Choudhary H., Berton P., Gurau G., Myerson A. S., Rogers R. D. (2018). Chem. Commun..

[cit49] Doherty S., Knight J. G., Backhouse T., Abood E., Alshaikh H., Clemmet A. R., Ellison J. R., Bourne R. A., Chamberlain T. W., Stones R., Warren N. J., Fairlamb I. J. S., Lovelock K. R. J. (2018). Adv. Synth. Catal..

[cit50] Li L., Wang J., Wu T., Wang R. (2012). Chem.–Eur. J..

[cit51] More S., Jadhav S., Salunkhe R., Kumbhar A. (2017). Mol. Catal..

[cit52] Gu Y., Favier I., Pradel C., Gin D. L., Lahitte J.-F., Noble R. D., Gómez M., Remigy J.-C. (2015). J. Membr. Sci..

[cit53] Patil J. D., Korade S. N., Patil S. A., Gaikwad D. S., Pore D. M. (2015). RSC Adv..

[cit54] Wang R., Twamle B., Shreeve J. B. (2006). J. Org. Chem..

[cit55] Adam M. S. S., Mohamad A. D., El-Hady O. M. (2014). Monatsh. Chem..

[cit56] Cao Y.-Z., Zhao H.-Y., Bai F.-Y., Xing Y.-H., Wei D.-M., Niu S.-Y., Shi Z. (2011). Inorg. Chim. Acta.

[cit57] Williams R. J. P. (1954). J. Phys. Chem..

[cit58] Einstein A., Podolsky B., Rosen N. (1953). Phys. Rev..

[cit59] Ramesh R., Maheswaran S. J. (2003). J. Inorg. Biochem..

[cit60] Pravin N., Raman N. (2013). Inorg. Chem. Commun..

[cit61] Adam M. S. S. (2015). Monatsh. Chem..

[cit62] Das U. K., Ben-David Y., Leitus G., Diskin-Posner Y., Milstein D. (2019). ACS Catal..

[cit63] Abdel-Rahman L. H., Abu-Dief A. M., Newair E. F., Hamdan S. K. (2016). J. Photochem. Photobiol., B.

[cit64] Jayaseelan P., Akila E., Rani M. U., Rajavel R. (2016). J. Saudi Chem. Soc..

[cit65] Abdel-Rahman L. H., Abu-Dief A. M., Basha M., Abdel-Mawgoud A. A. H. (2017). Appl. Organomet. Chem..

[cit66] Jash U., Chakraborty G., Sinha S., Sikari R., Mondal R., Paul N. D. (2018). Asian J. Org. Chem..

[cit67] El-Baradie K. Y., El-Wakiel N. A., El-Ghamry H. A. (2015). Appl. Organomet. Chem..

[cit68] Abdel-Rahman L. H., Abu-Dief A. M., El-Khatib R. M., Abdel-Fatah S. M. (2016). J. Photochem. Photobiol., B.

[cit69] Ramakrishna V., Reddy N. D. (2017). Dalton Trans..

[cit70] Kong F., Zhou C., Wang J., Yu Z., Wang R. (2013). ChemPlusChem.

[cit71] Kolychev E. L., Asachenko A. F., Dzhevakov P. B., Bush A. A., Shuntikov V. V., Khrustalevc V. N., Nechaev M. S. (2013). Dalton Trans..

[cit72] Fortun S., Beauclair P., Schmitzer A. R. (2017). RSC Adv..

[cit73] Dakarapu R., Falck J. R. (2018). J. Org. Chem..

[cit74] Neely J. M., Bezdek M. J., Chirik P. J. (2016). ACS Cent. Sci..

[cit75] Vijjamarri S., Streed S., Serum E. M., Sibi M. P., Du G. (2018). ACS Sustainable Chem. Eng..

[cit76] You L.-X., Liu H.-J., Cui L.-X., Ding F., Xiong G., Wang S.-J., Ren B.-Y., Dragutan I., Dragutan V., Sun Y.-G. (2016). Dalton Trans..

[cit77] Valdés H., García-Eleno M. A., Canseco-Gonzalez D., Morales-Morales D. (2018). ChemCatChem.

[cit78] Mastalir M., Pittenauer E., Stöger B., Allmaier G., Kirchner K. (2017). Org. Lett..

[cit79] Halgren T. A. (1996). J. Comput. Chem..

