# Supplementary material for: Biological and catalytic potential of sustainable low and high valent metal-Schiff base sulfonate salicylidene pincer complexes
Source: RSC Adv. 2019 Oct 25;9(59):34311–29. doi: 10.1039/c9ra06816c (PMC9074042; doi:10.1039/c9ra06816c)
Supplement: RA-009-C9RA06816C-s001 [file RA-009-C9RA06816C-s001.pdf]

Electronic Supplementary Material (ESI) for RSC Advances.
This journal is © The Royal Society of Chemistry 2019

Biological and catalytic potential of sustainable low and high valent metal-Schiff base sulfonate salicylidene pincer complexes

Mohamed Shaker S. Adam[1,2]*, Omar M. El-Hady[2], Farman Ullah[3]

[1] Department of Chemistry, College of Science, King Faisal University, P.O. Box 380 Al Hufuf 31982 Al Hassa, Saudi Arabia.

[2] Chemistry Department, Faculty of Science, Sohag University, Sohag-82534, Egypt.

[3] Department of Chemistry, Winnipeg University 515 Portage Avenue, Winnipeg, Manitoba, R3B 2E9 Canada

*corresponding author e-mail: madam@kfu.edu.sa; shakeradam61@yahoo.com (M.S.S. Adam).

TABLE S1

Activity index (%) of the antibacterial assay for HSaln ligand and MSaln complexes.

	Activity index (%)		
Compound	Serratia Marcescence (-ve)	Escherichia coli (-ve)	Staphylococcus aureus (+ve)
HSaln	25.00	21.62	30.43
VOSaln	90.00	91.89	91.30
VOSaln-Ph	75.00	78.38	78.26
MnSaln	85.00	89.19	86.96
MoO_2Saln	82.50	86.49	82.61
UO_2Saln	95.00	94.59	95.65

TABLE S2

Activity index (%) of antifungal assay for HSaln ligand and MSaln complexes.

	Activity index (%)		
Compound	Candida albicans	Aspergillus flavus	Trichophyton Rubrum
HSaln	18.92	32.00	35.48
VOSaln	78.38	76.00	80.65
VOSaln-Ph	91.89	92.00	93.55
MnSaln	89.19	88.00	90.32
MoO_2Saln	86.49	84.00	87.09
UO_2Saln	97.30	96.00	96.77

Table S3

Cytotoxic activity (IC_{50}) of HSaln ligand and its MSaln complexes various Colon carcinoma cells, (HCT-116 cell line), hepatic cellular carcinoma cells (HepG-2) and breast carcinoma cells (MCF-7).

	IC_{50} (μg μL^{-1})		
Compound	HCT-116	MCF-7	HepG-2
HSaln ligand	111.50±0.14	85.70±0.23	93.20±0.15
MnSaln	23.50±0.12	14.20±0.12	17.50±0.17
VOSaln-Ph	35.60±0.21	24.90±0.13	28.90±0.15
VOSaln	29.20±0.13	18.50±0.11	21.30±0.12
MoO_2Saln	33.10±0.15	21.30±0.16	25.40±0.18
UO_2Saln	17.70±0.19	9.60±0.10	11.60±0.13
Vinblastine standard	13.30±0.11	4.12±0.14	7.50±0.10

NMR spectral data of HSaln ligand:

^{1}H NMR (DMSO-d_6, 400 MHz): δ 6.50 (t, ^3J = 6.9 and 7.2 Hz, 1H), 6.74 (d, ^3J = 8.2 Hz, 1H), 6.94 (dd, ^3J = 8.0 and 8.3 Hz, 1H), 7.22 (t, ^3J = 7.0 and 7.4 Hz, 1H), 7.70 (dd, ^3J = 7.7 Hz, 2H), 7.92 (s, 1H), 10.26 (s, 1H, phenolic-OH), 10.82 ppm (s br, 1H, CH=N).

^{13}C NMR (100 MHz, DMSO-d_6, dept-135): δ 110.18 (C_q), 115.05 (CH), 116.83 (CH), 116.96 (CH), 121.48 (CH), 126.92 (CH), 131.61 (CH), 134.20 (CH), 140.72 (C_q), 151.94 (C_q), 161.15 (C_q), 169.99 (C_q), 192.11 ppm (CH, CH=N).

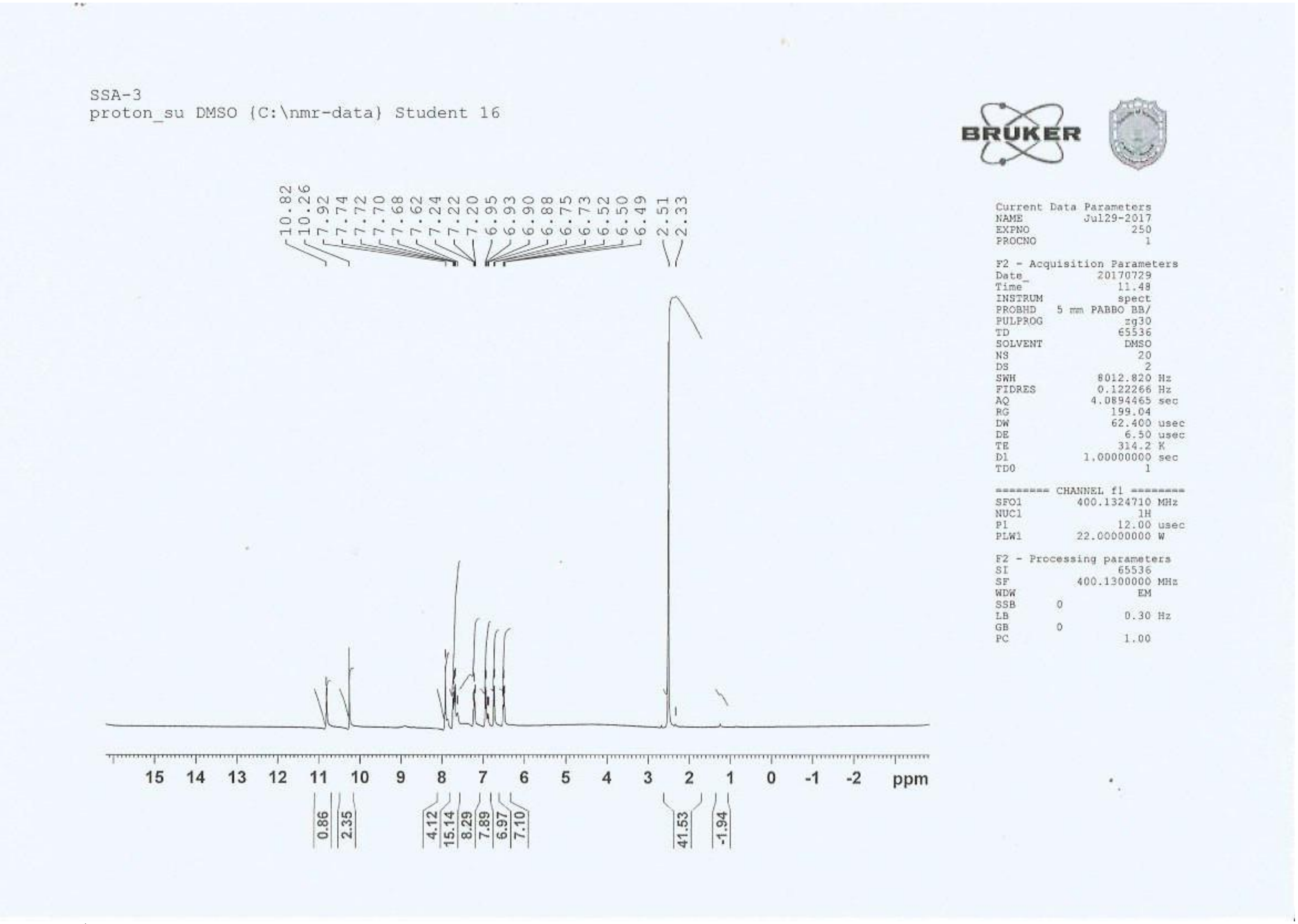

Fig. S1: ^{1}HNMR of HSaln ligand.

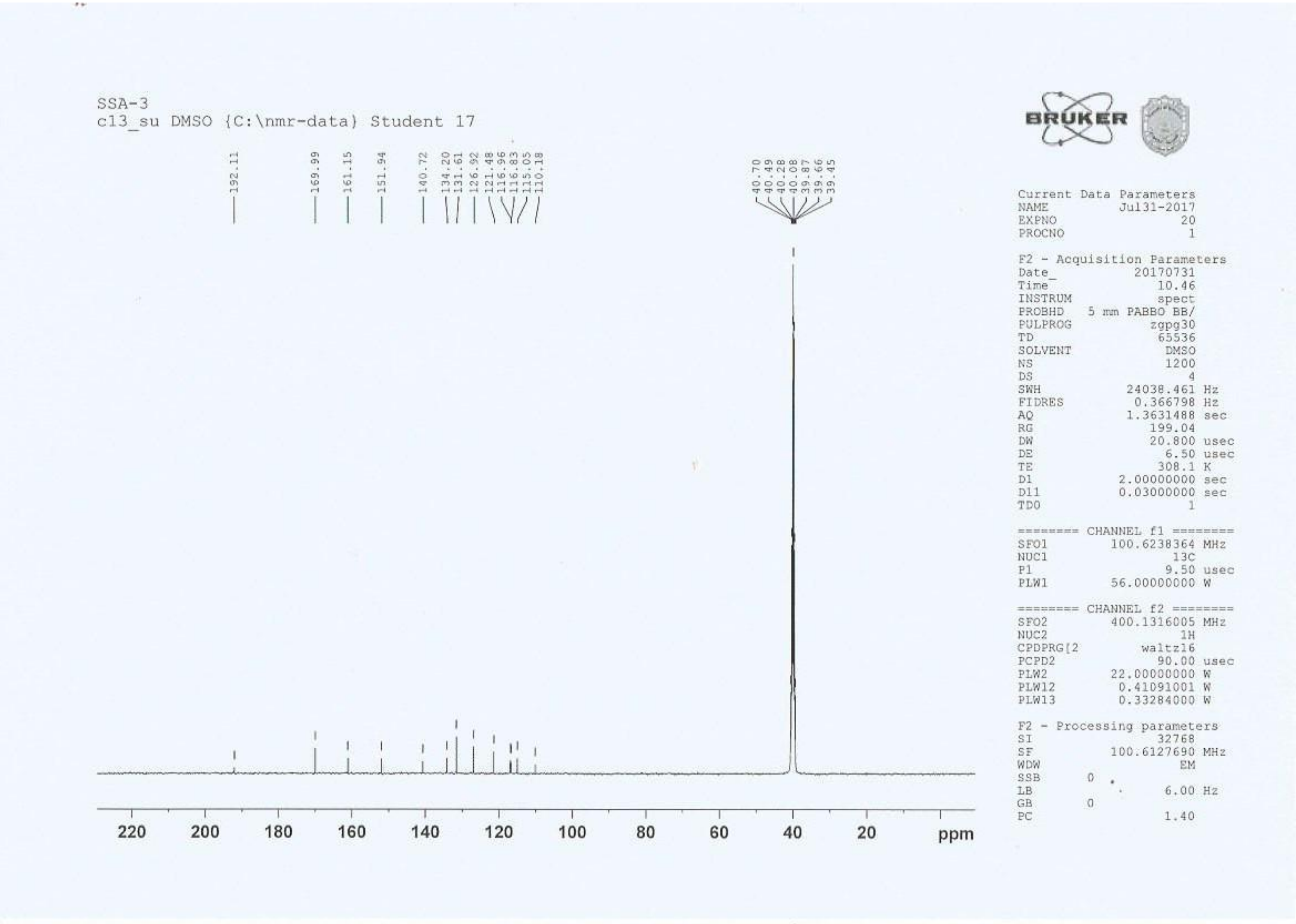

Fig. S2: ^{13}CNMR of HSaln ligand.

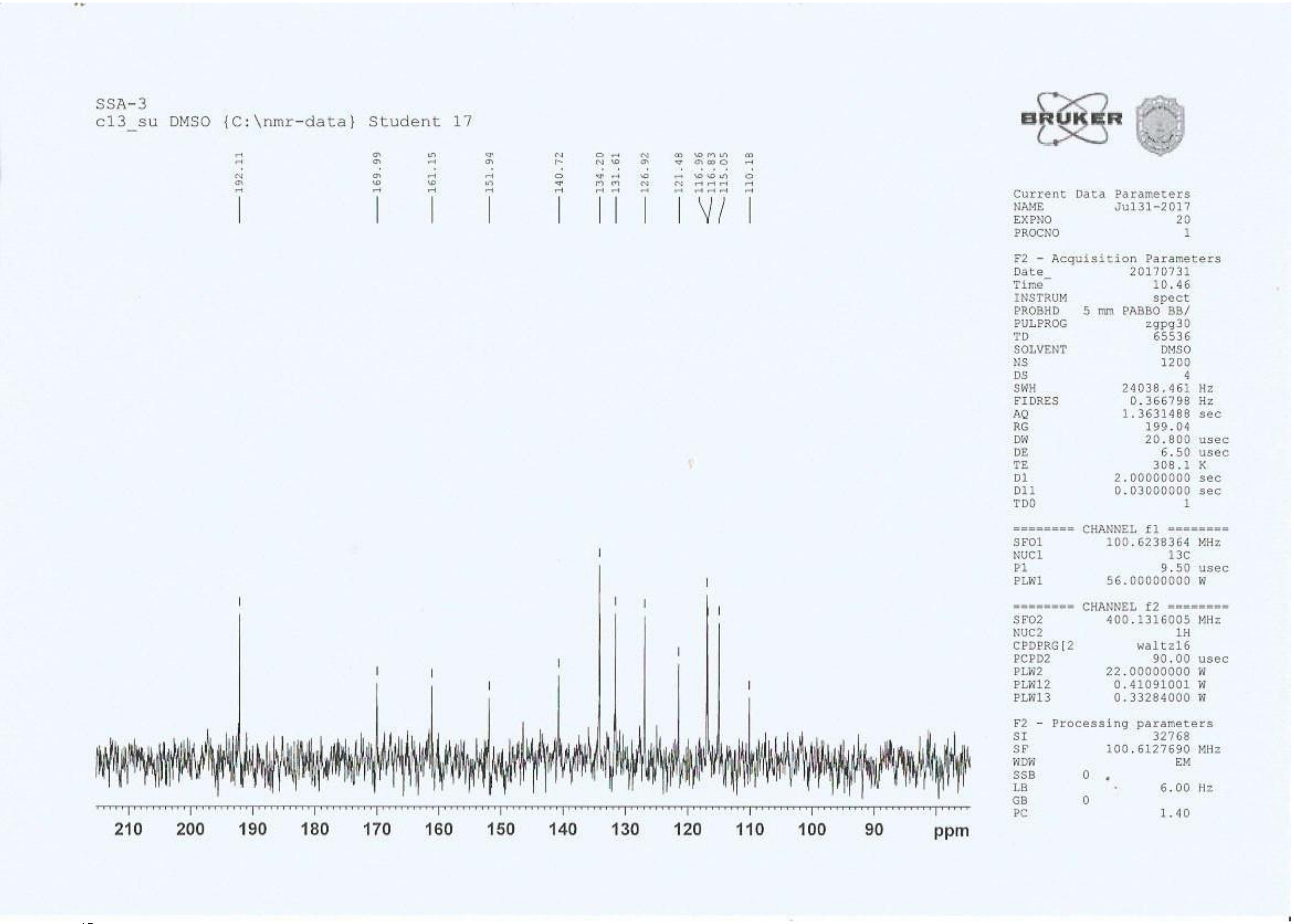

Fig. S3: ^{13}CNMR of HSaln zoom in the aromatic region.

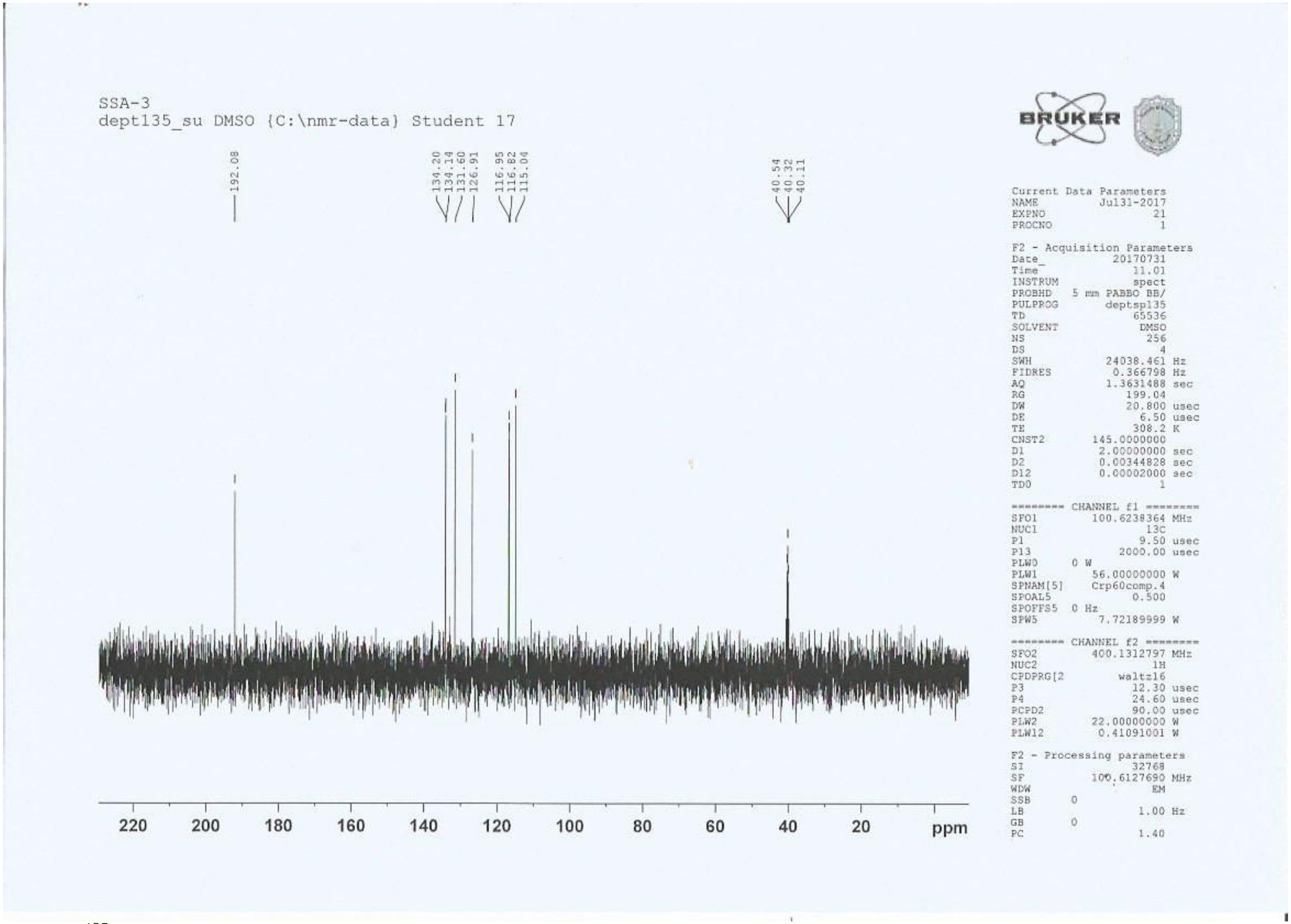

Fig. S4: [135]Dept CNMR of HSaln ligand.

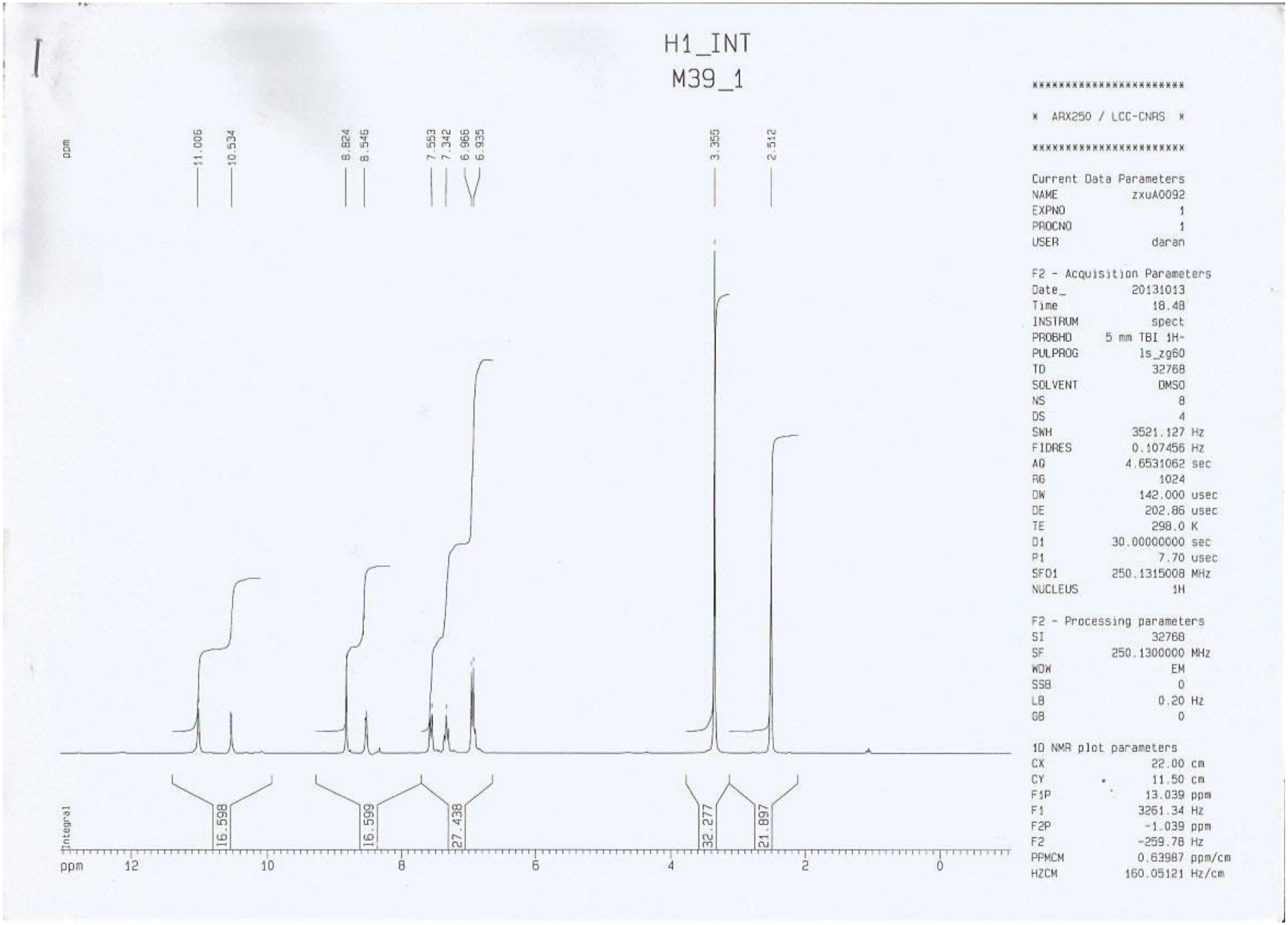

Fig. S5: ^{1}HNMR of MoO_2Saln complex.

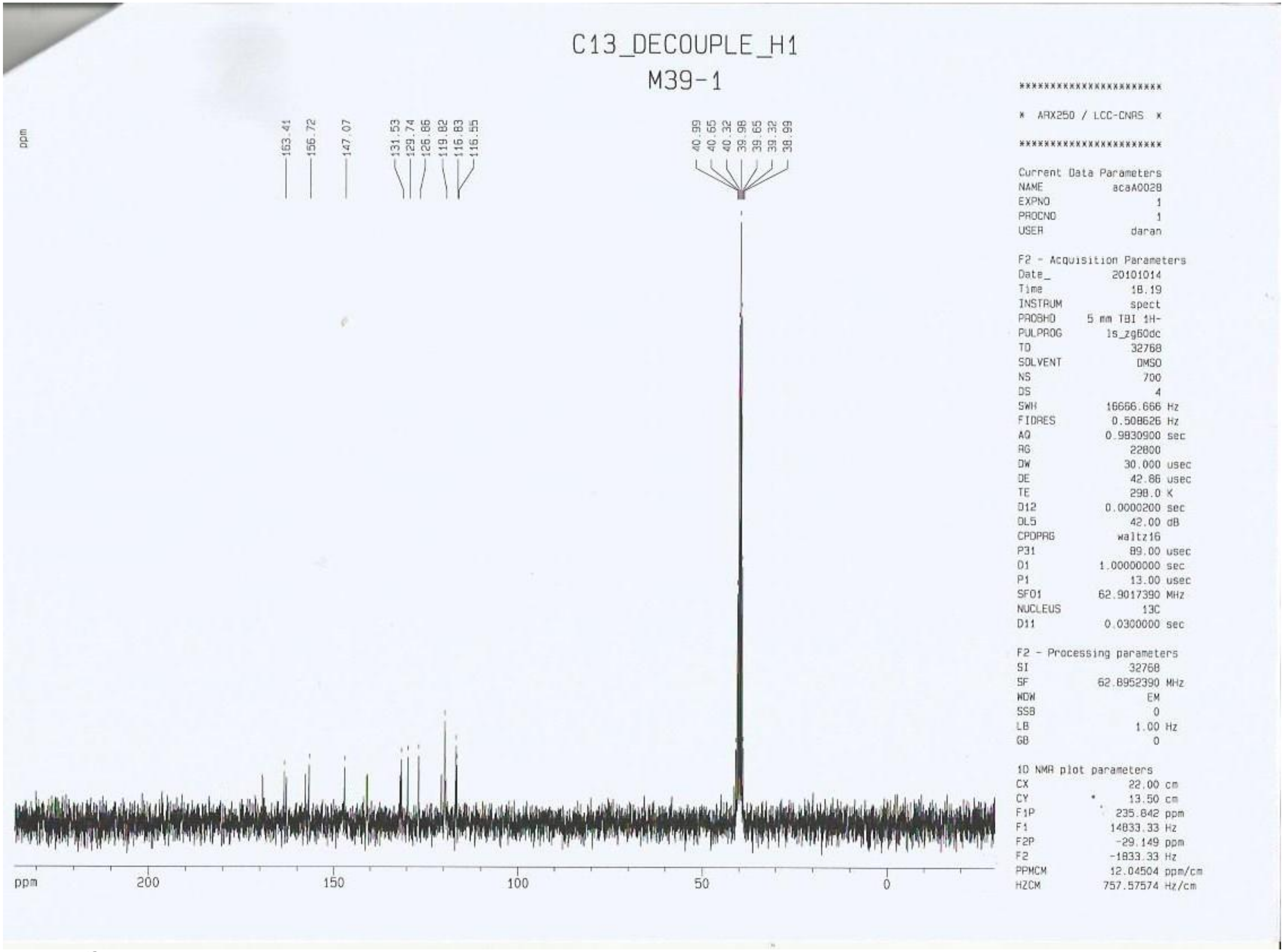

Fig. S6: ^{13}CNMR of MoO_2Saln complex.

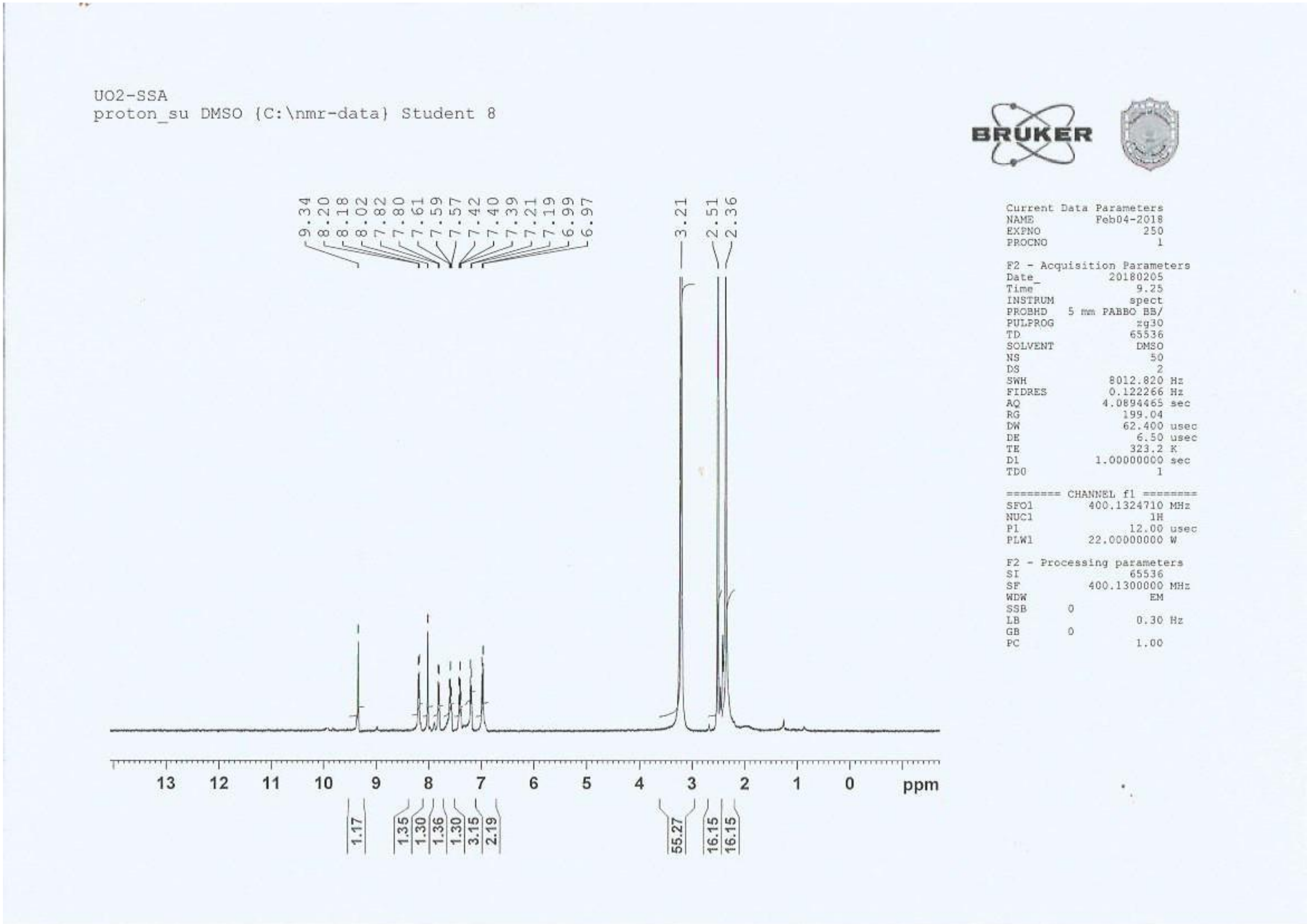

Fig. S7: ^{1}HNMR of UO_2Saln complex.

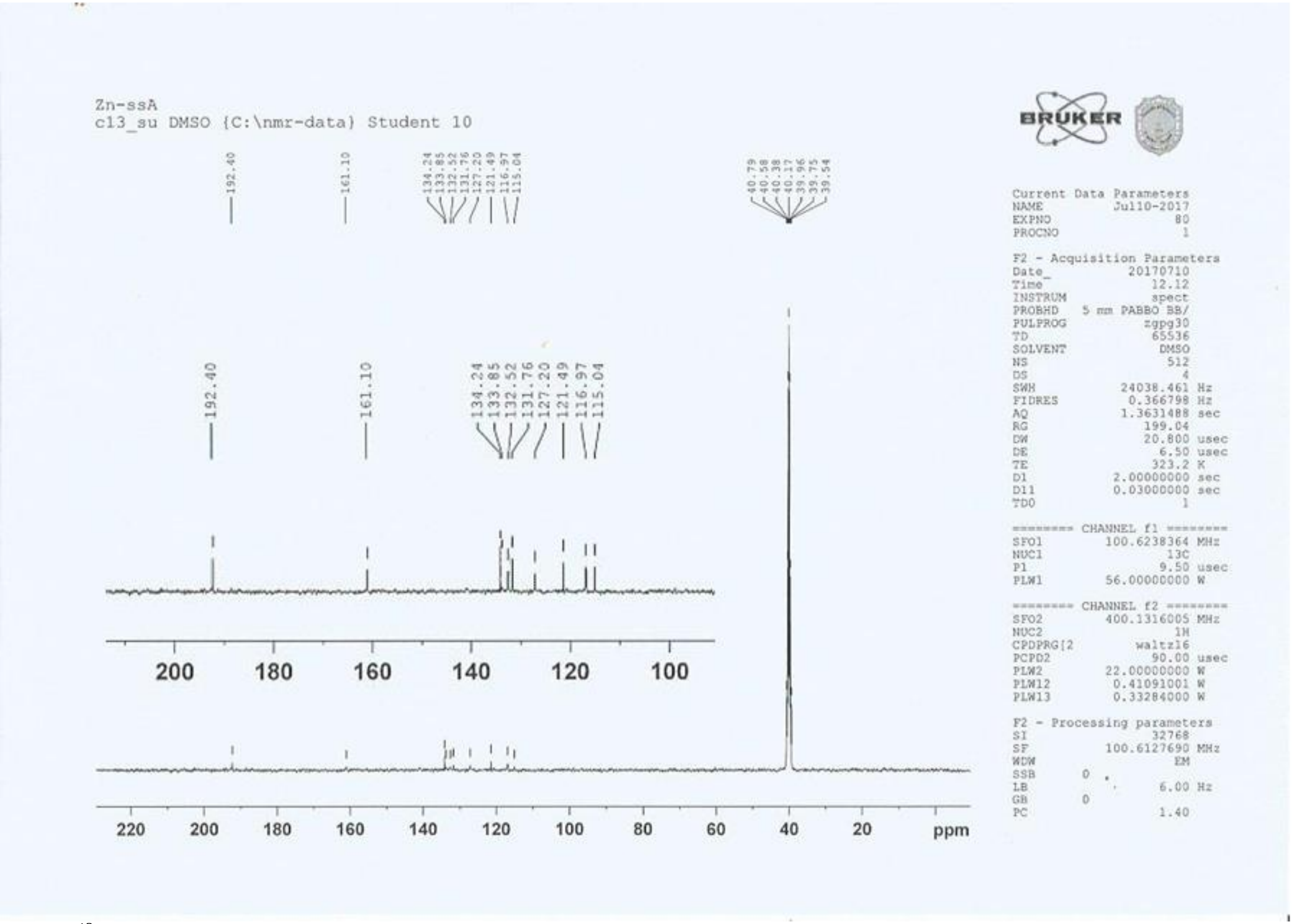

Fig. S8: ^{13}CNMR of UO_2Saln complex.

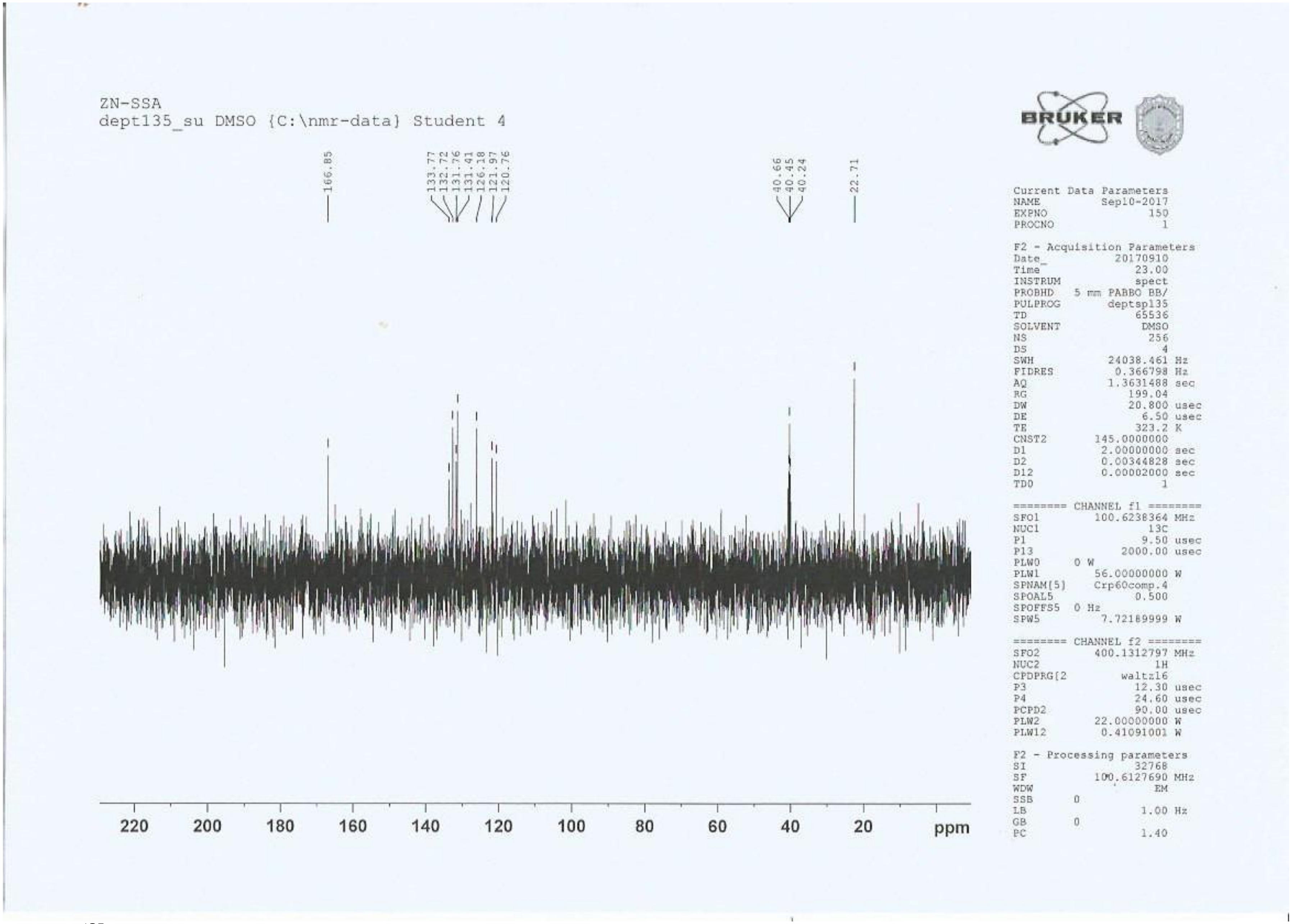

Fig. S9: ^{135}Dept CNMR of UO_2Saln complex.

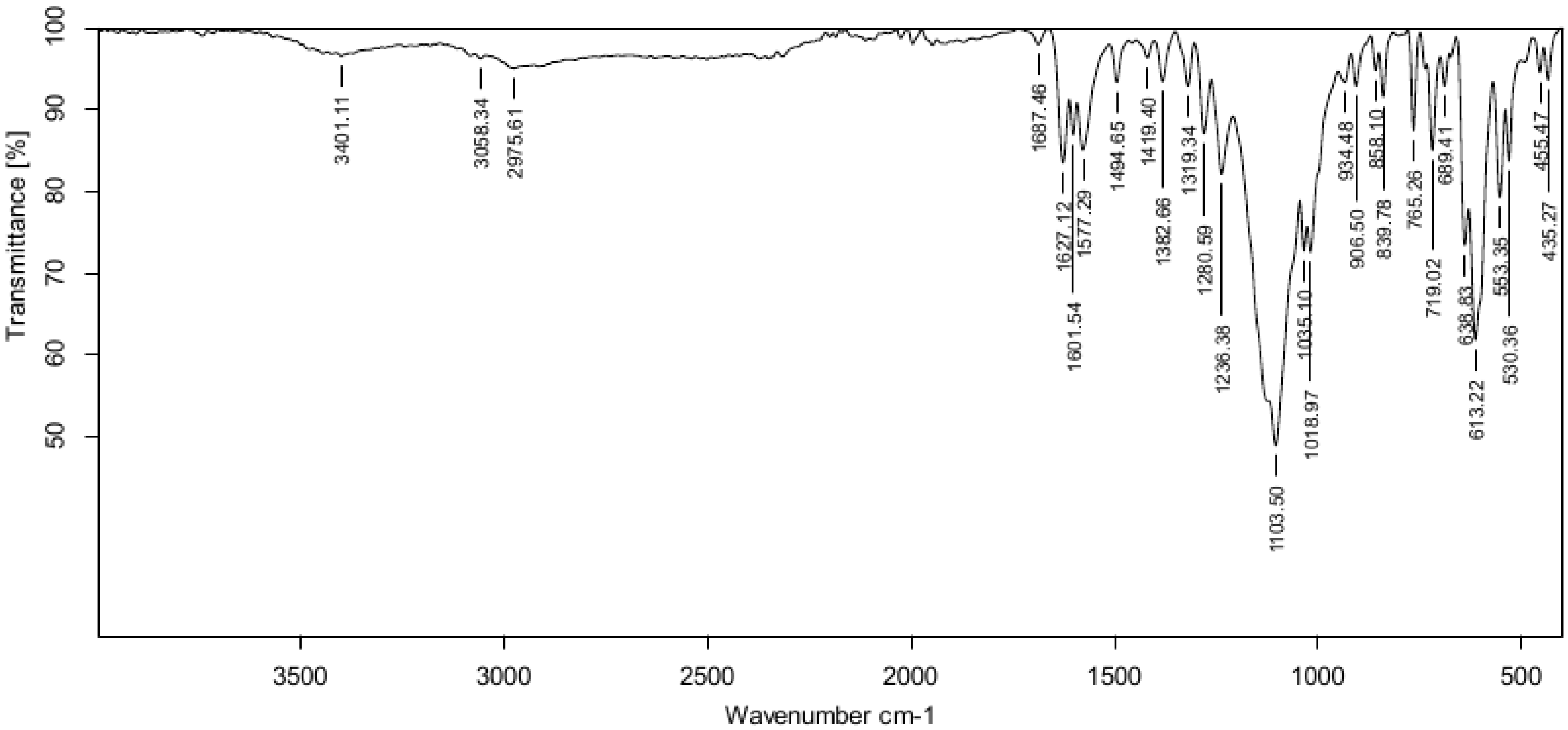

Fig. S10: IR spectra of HSaln ligand.

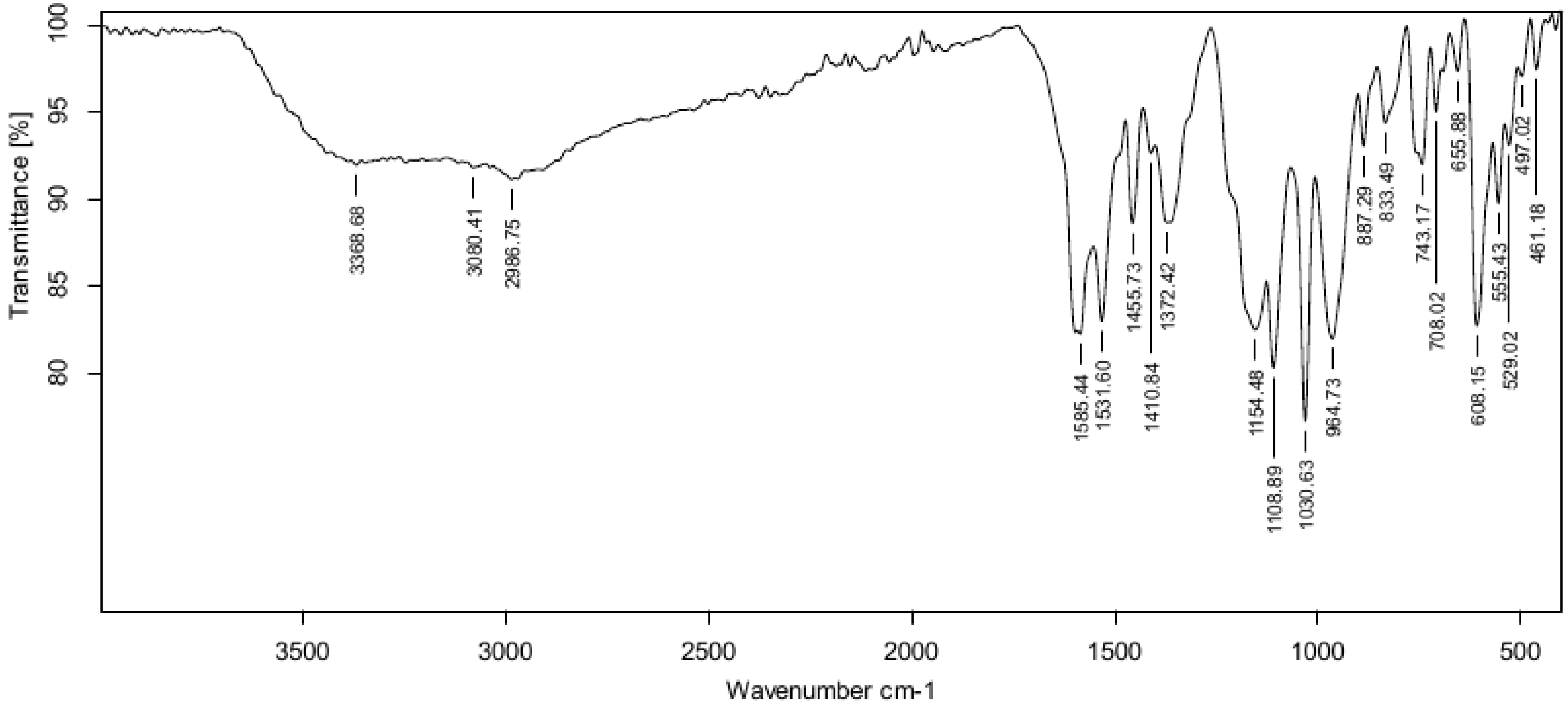

Fig. S11: IR spectra of VOSaln complex.

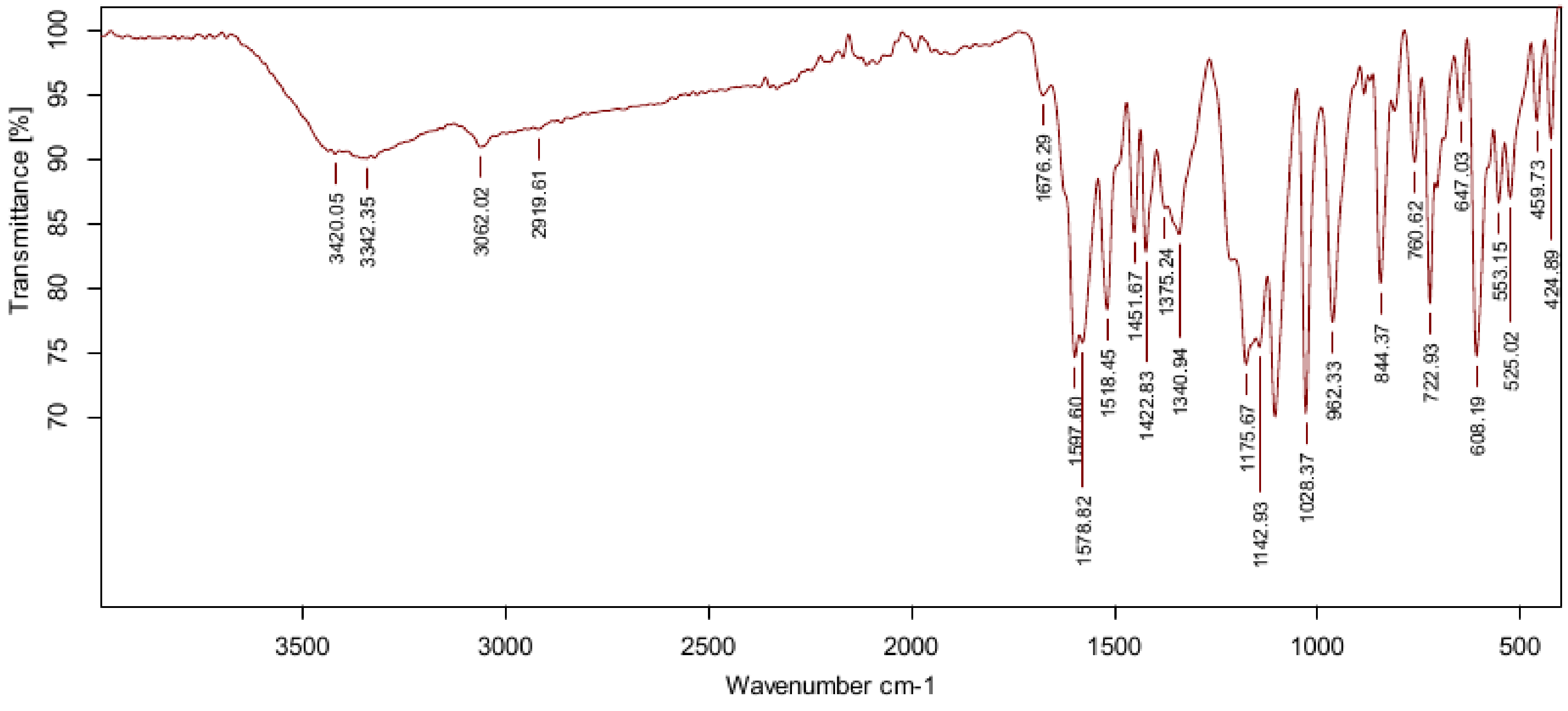

Fig. S12: IR spectra of VOSaln-Ph complex.

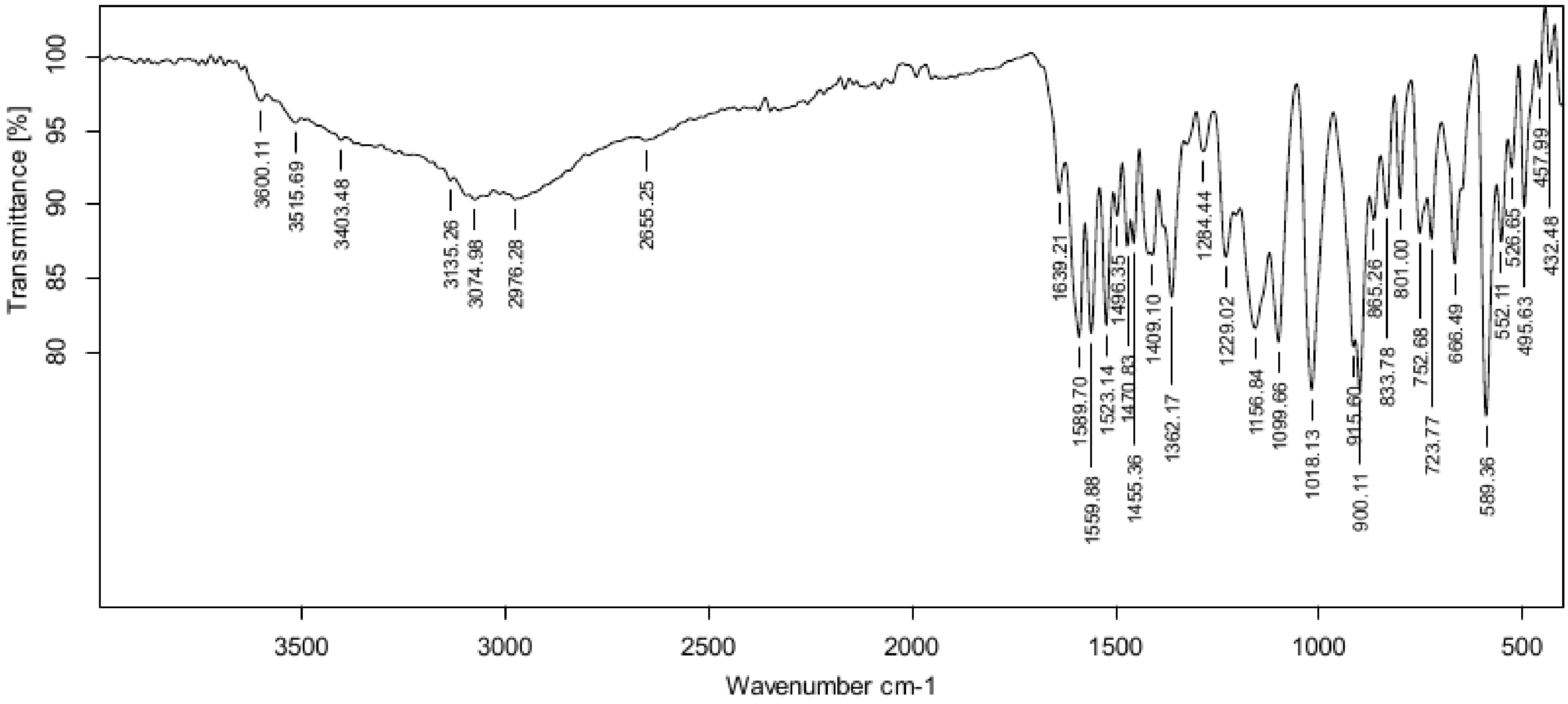

Fig. S13: IR spectra of UO_2Saln complex.

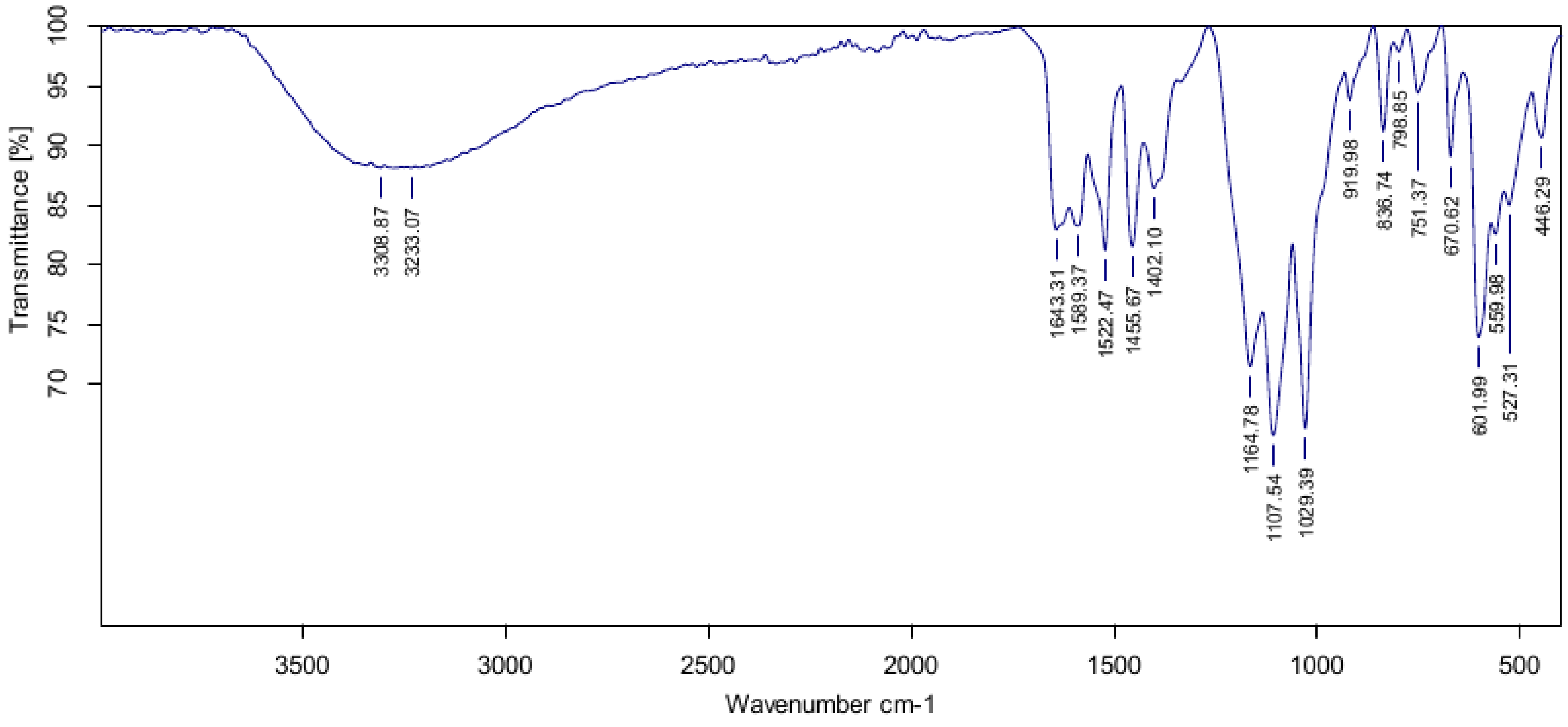

Fig. S14: IR spectra of MnSaln complex.

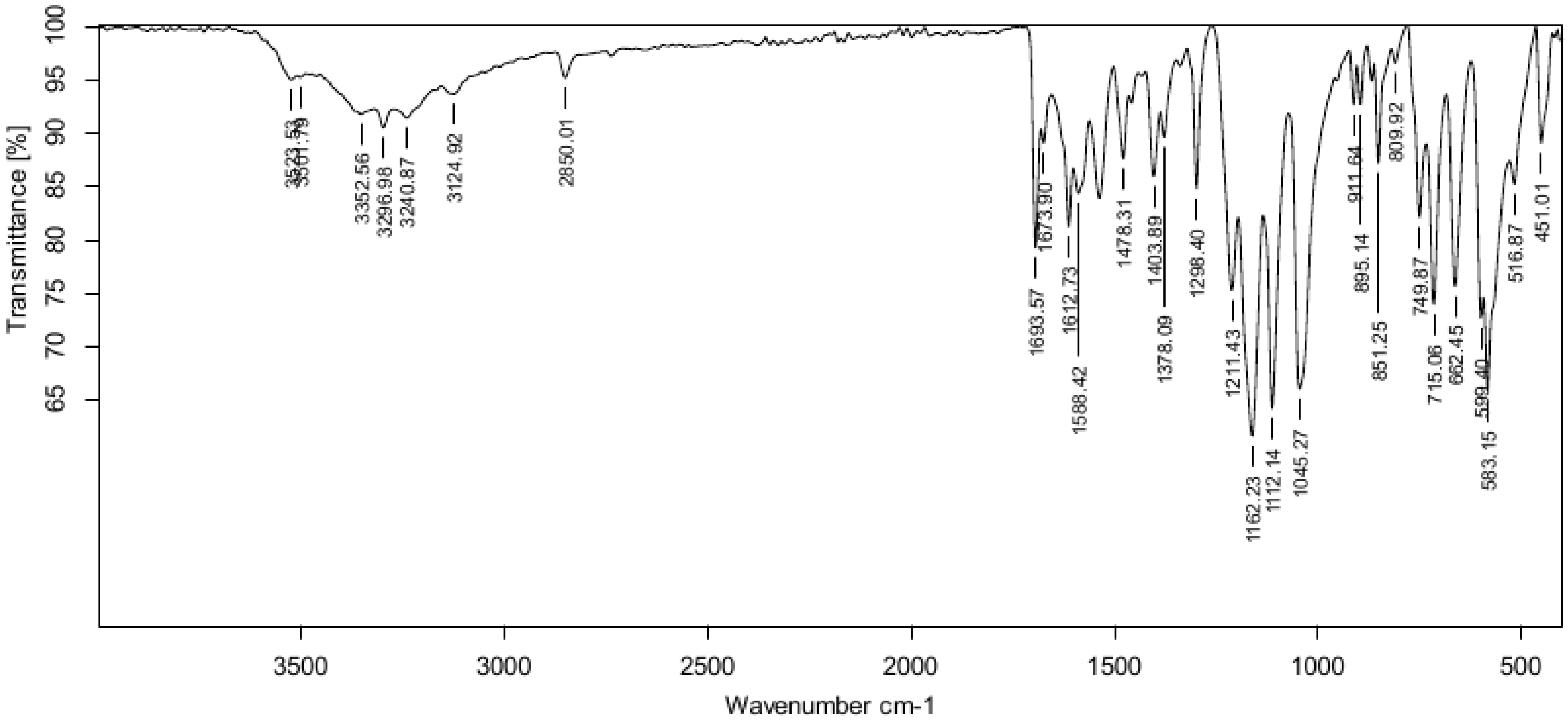

Fig. S15: IR spectra of MoO_2Saln complex.

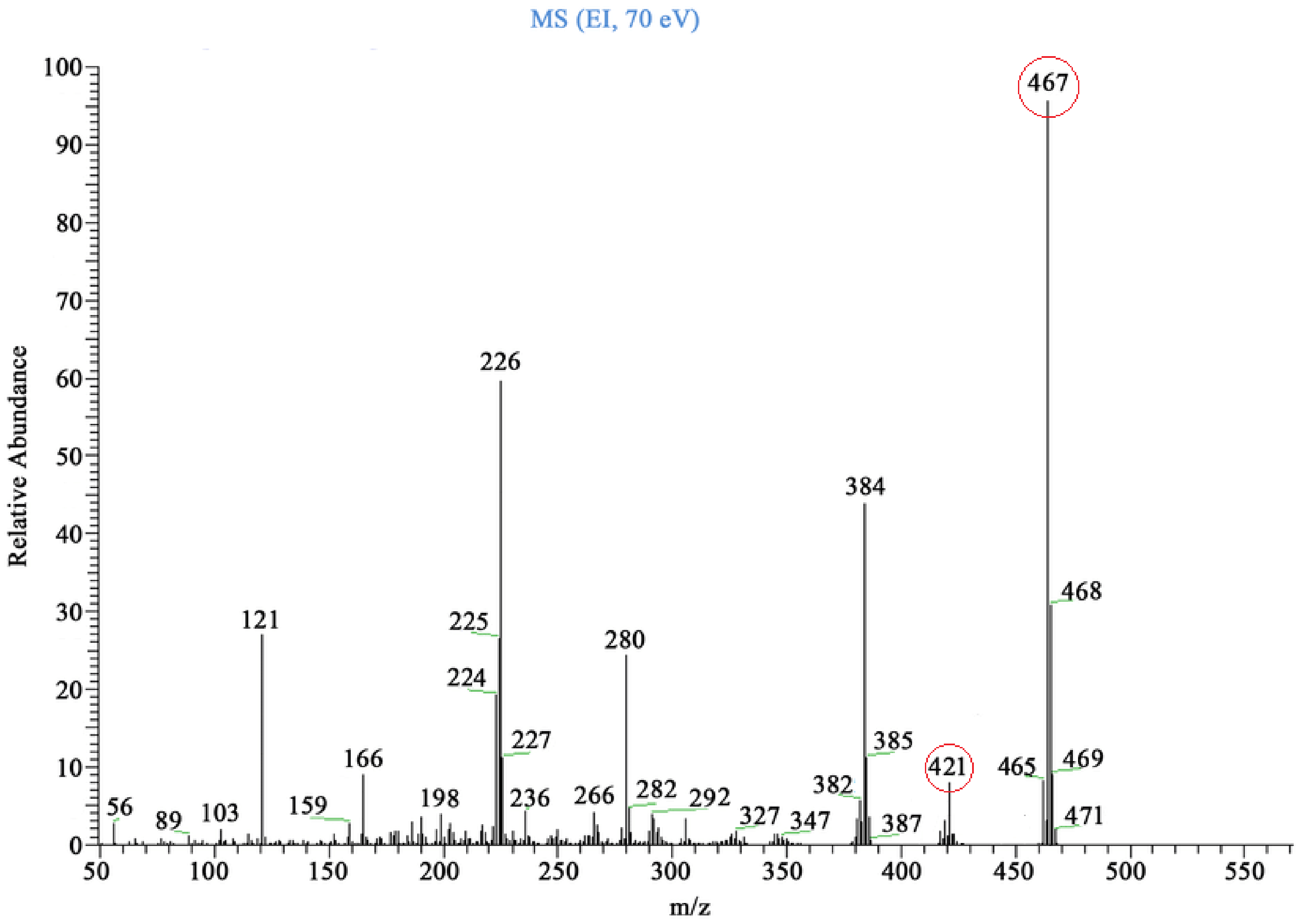

Fig. S16: MS of VOSaln complex.

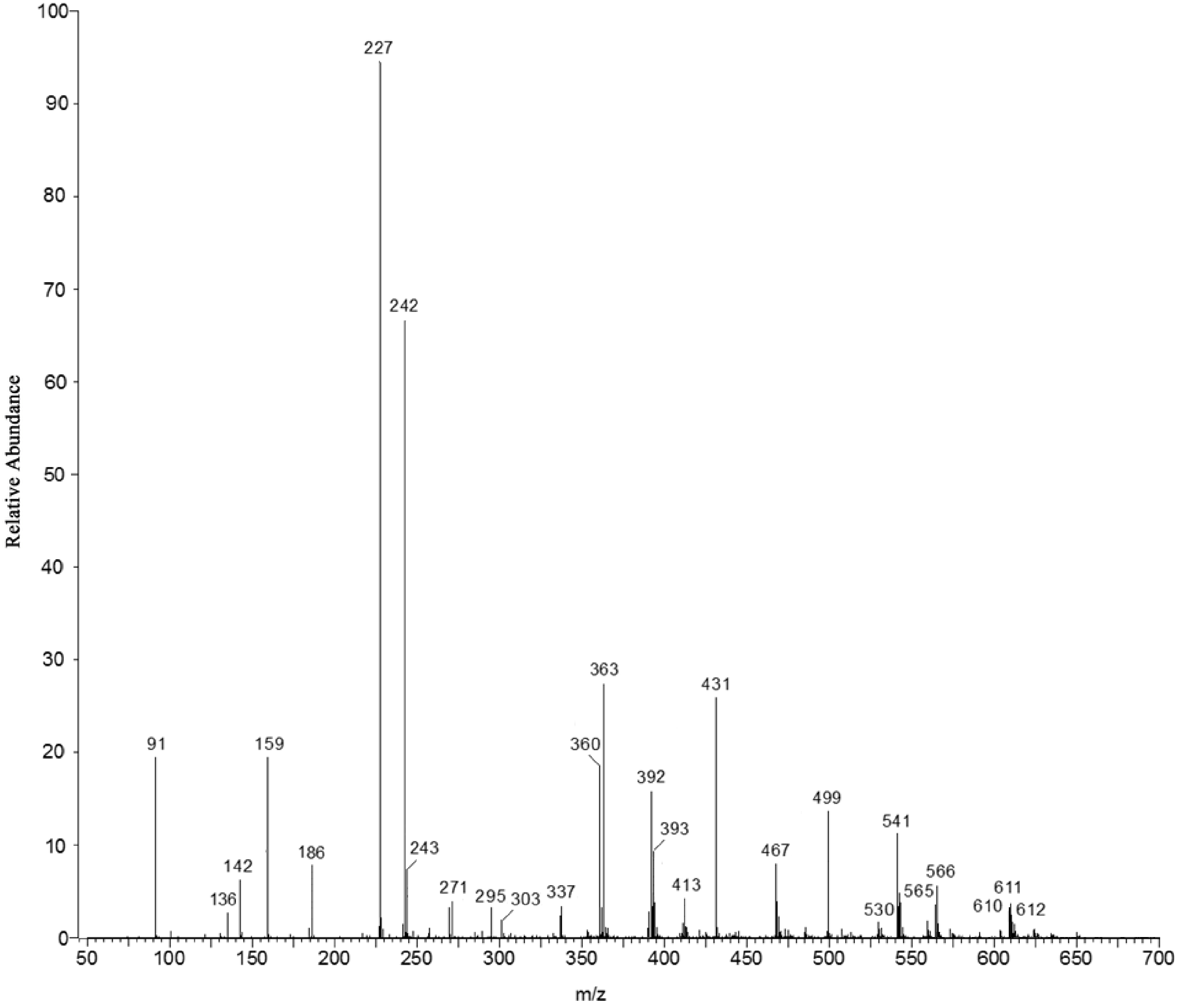

Fig. S17: MS of VOSaln-Ph complex.

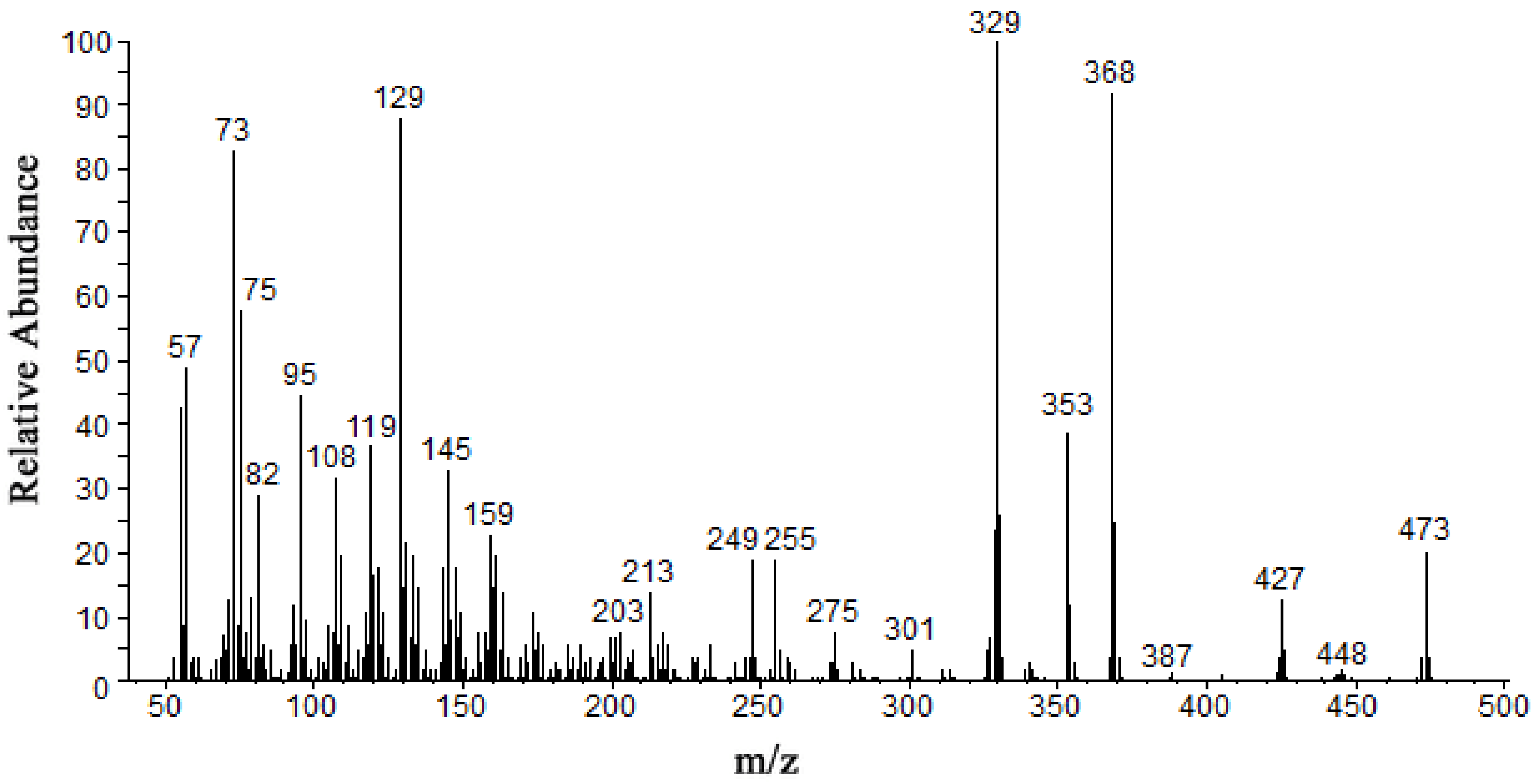

Fig. S18: MS of MnSaln complex.

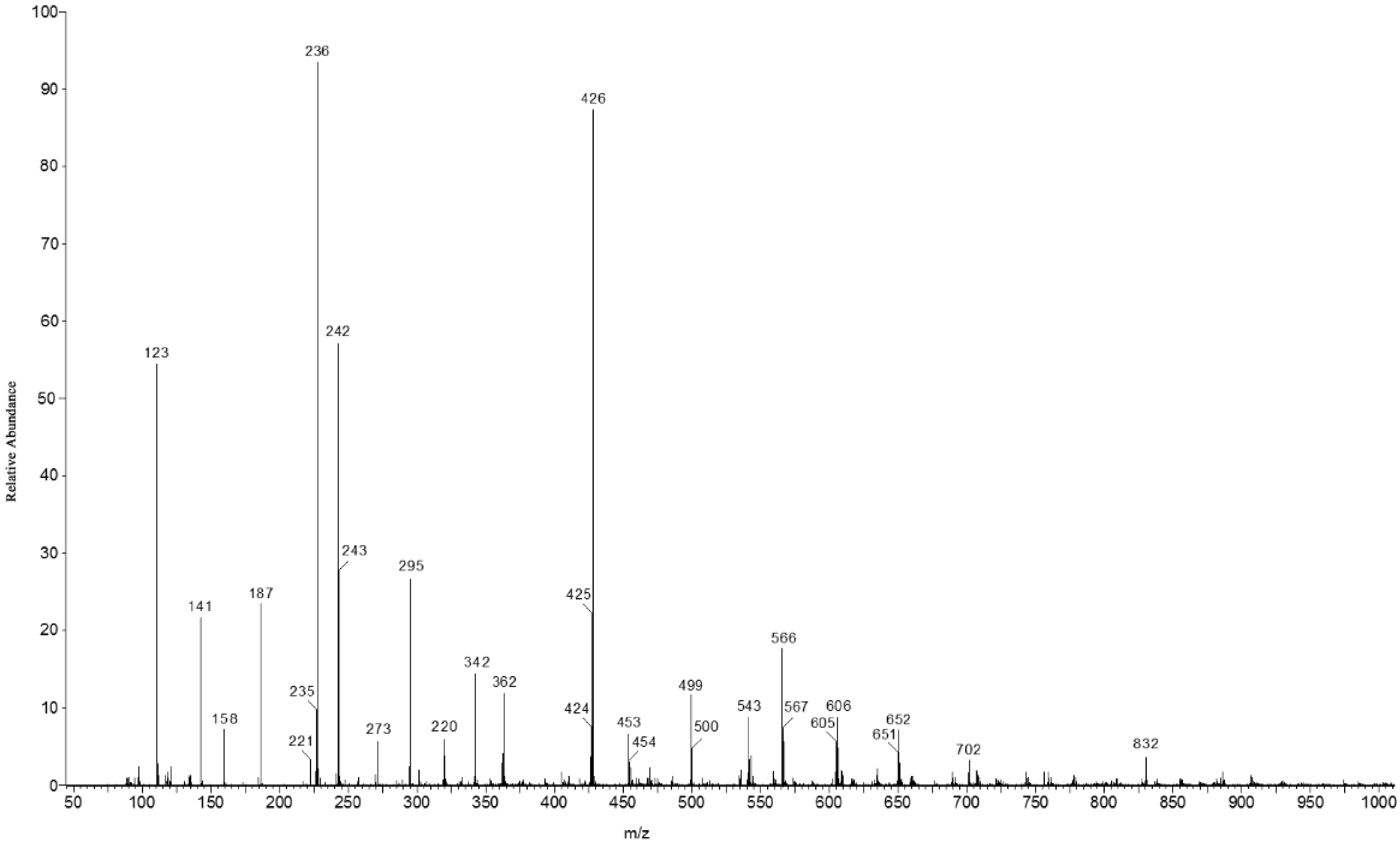

Fig. S19: MS of UO_2Saln complex.

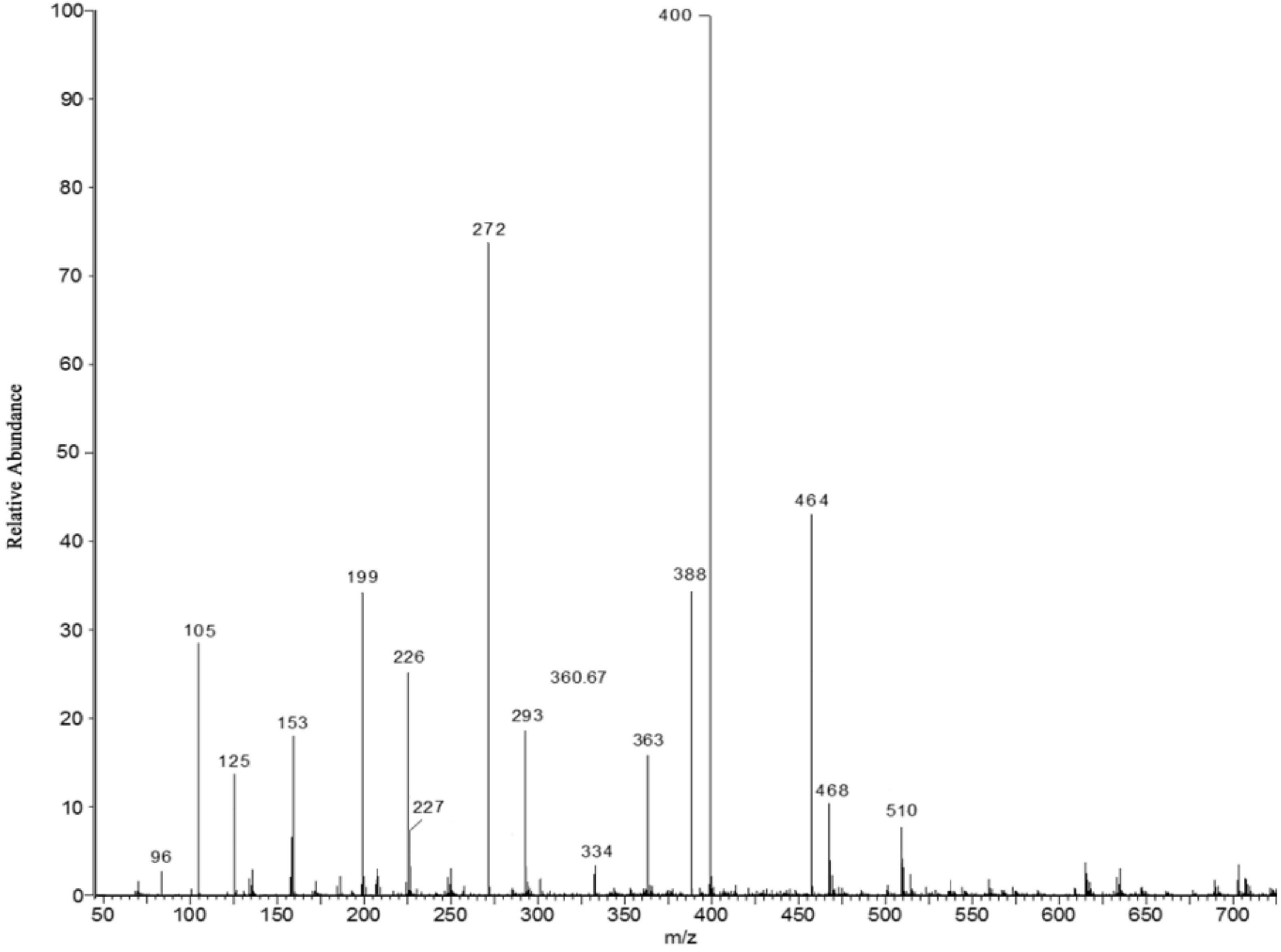

Fig. S20: MS of MoO_2Saln complex.

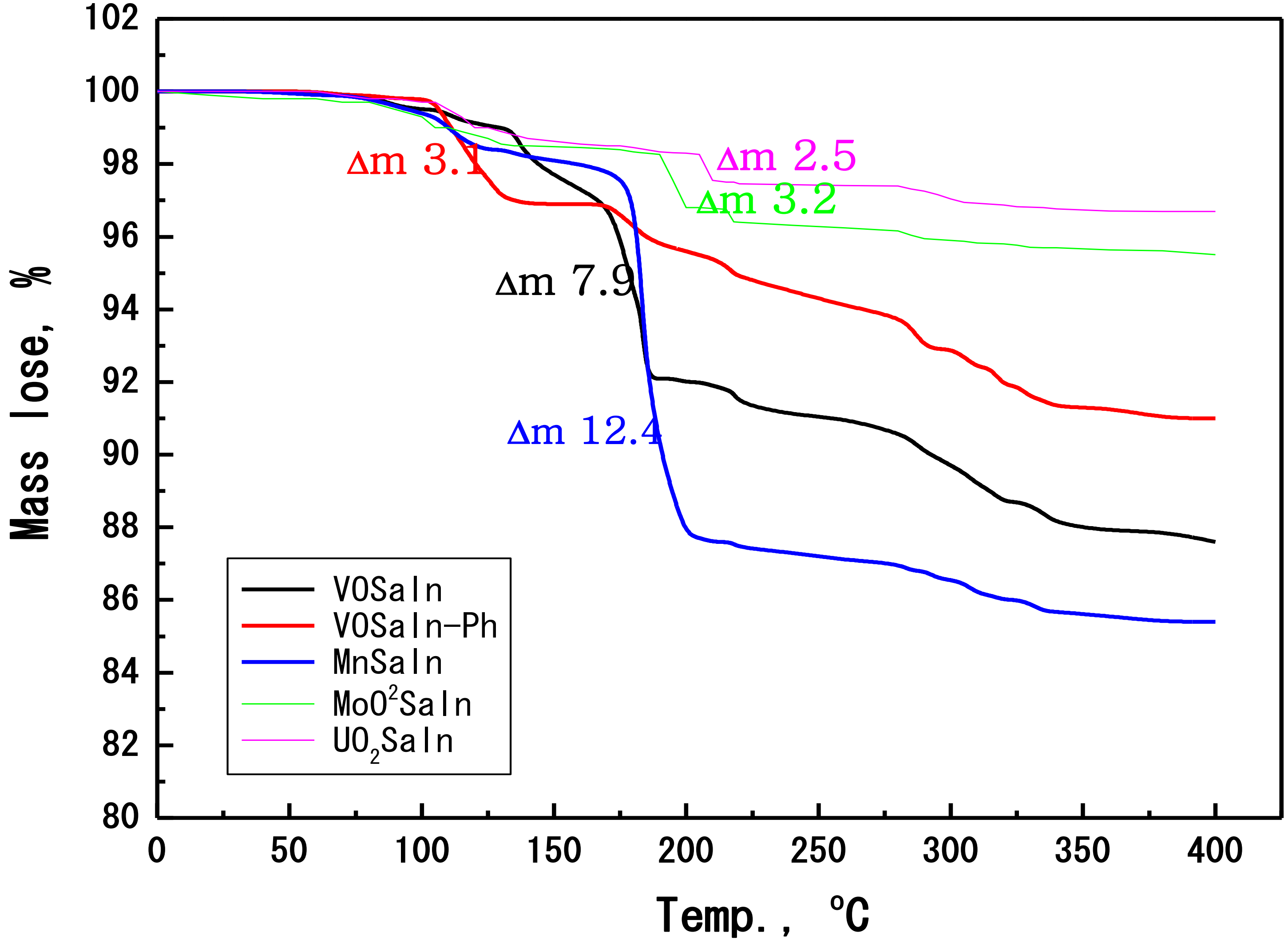

Fig. S21: TGA of MSaln complexes.

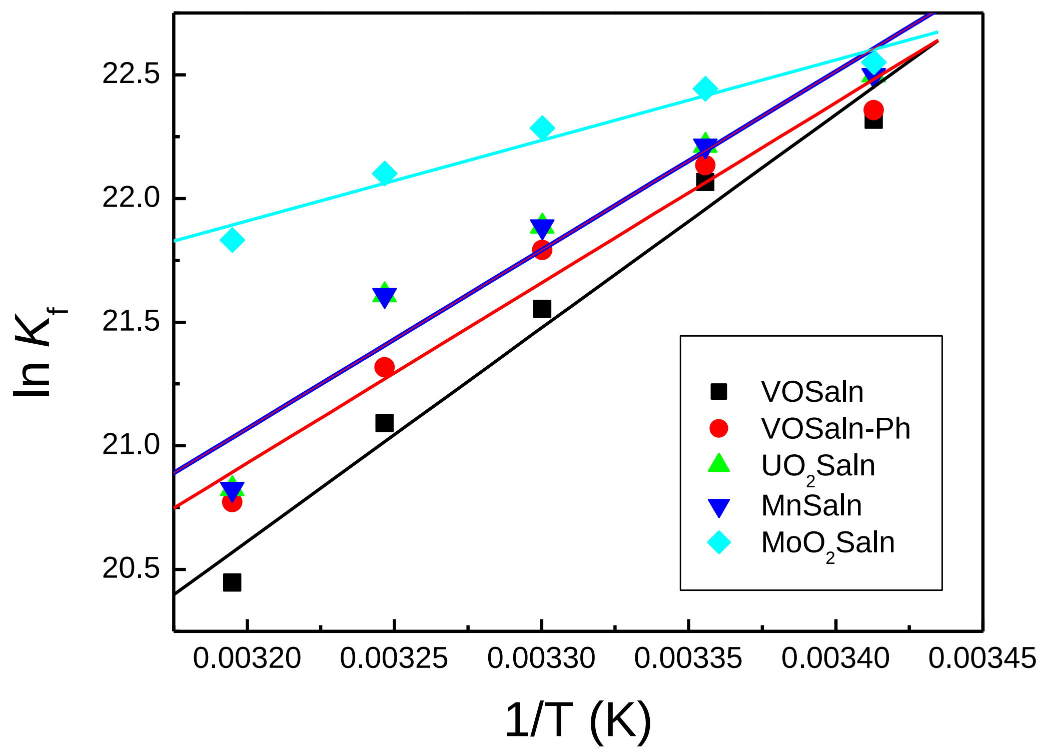

Fig. S22. Determination of thermodynamic parameters of MSaln complexes formation from ln K_f values.
